# Codon-aware multi-scale feature fusion for metagenomic sequence classification

**DOI:** 10.1371/journal.pone.0359090

**Published:** 2026-10-05

**Authors:** Yan Qian, Quanjie Song, Xinyi Xie, Li Deng

**Affiliations:** 1 School of Mechatronic Engineering and Automation, Shanghai University, Shanghai, People’s Republic of China; 2 Shanghai Key Laboratory of Power Station Automation Technology, Shanghai, People’s Republic of China; University of Enna Kore: Universita degli Studi di Enna ‘Kore’, ITALY

## Abstract

Microorganisms exert profound influences on both natural ecosystems and human society. As the cornerstone of microbial research, metagenomics relies on the precise analysis of large-scale, multi-source genomic data. However, discriminating among diverse sequence types remains a formidable challenge. To address this, we present CamFi (Codon-aware Multi-scale Feature Fusion), a unified framework integrating overlapping nucleotide-triplet representations with multi-scale dilated convolutions for classifying prokaryotic chromosomes, eukaryotic chromosomes, plasmids, and viruses. Benchmark evaluations demonstrate strong mean F1 scores of 97.70% for eukaryotic chromosomes and 94.13% for plasmids. On the CAMI II marine dataset, CamFi achieved a weighted F1 of 82.65%, ranking second among six methods based on reported aggregate metrics. In a balanced four-class benchmark, CamFi attained a macro-F1 of 93.77%, compared with DeepMicroClass (90.03%) and the XGBoost stage of 4CAC (64.66%). These results establish CamFi as a competitive approach for unified metagenomic contig classification under the evaluated conditions.

## Introduction

Microorganisms play an indispensable role in driving the biogeochemical cycling of elements across Earth’s diverse ecosystems [1,2]. Microbial communities are complex assemblages comprising multiple biological entities, including not only cellular organisms that carry ribosome-encoding genes and those capable of encoding capsid proteins, but also autonomous replicons such as viruses and plasmids that depend on hosts for replication [3]. As key extrachromosomal genetic elements, viruses and plasmids profoundly shape the diversity and function of microbial communities by mediating the horizontal transfer of genetic material between and within microorganisms. These mobile genetic units, together with transposable elements, are collectively referred to as mobile genetic elements (MGEs).

The compositional complexity of microbial communities varies enormously, ranging from assemblages dominated by a few abundant strains to those comprising thousands of distinct species, depending on the environment, timing, and methodology of metagenomic sampling. The small subunit rRNA gene (SSU) has been established as a universal phylogenetic marker owing to its high degree of conservation [4], making high-throughput sequencing of SSU amplicons a routine approach for assessing environmental microbial diversity [5,6]. In addition, by constructing clone libraries from natural microbiomes and performing random metagenomic sequencing, researchers can further explore the coding potential and genomic characteristics of uncultivated microorganisms, thereby inferring their functional roles within the community [6–14]. The explosive growth of metagenomic data has, on the one hand, created unprecedented opportunities for elucidating complex microbial interaction networks and the evolutionary dynamics of individual species [15,16]. On the other hand, efficiently and accurately reconstructing microbial genomes and MGEs from vast pools of mixed sequences has emerged as a pressing technical challenge. To address this, a common strategy is to partition metagenomic contigs into taxonomic groups, thereby reducing data complexity.

Existing tools for metagenomic contig classification can be broadly divided into two categories: supervised classification methods and unsupervised clustering methods [17]. Among the targets of such analyses, the identification of viral sequences represents a major focus. Viruses are ubiquitously distributed across aquatic, terrestrial, and host-associated ecosystems and are regarded as the most abundant biological entities on Earth [18,19]. In marine environments in particular, virus-mediated lysis significantly regulates the flow of carbon and energy toward lower trophic levels, exerting a profound influence on global biogeochemical cycles [20,21].

VirSorter [22] and VirFinder [23] were among the early representative tools for identifying viral contigs from metagenomic assemblies. The former classifies predictions into three confidence tiers based on virus-specific signals, whereas the latter employs k-mer frequencies coupled with a logistic regression model and demonstrates superior performance on short sequences that lack canonical viral gene markers [23]. Inspired by the success of k-mer-based strategies, deep learning approaches were subsequently introduced to viral sequence identification, giving rise to tools such as DeepVirFinder [24] and PPR-Meta [25]. These methods typically convert DNA sequences into one-hot encoded matrices and train neural networks to discriminate between virus- and host-derived contigs. PPR-Meta further integrates information from both nucleotide and codon pathways, enabling simultaneous classification of viruses, host chromosomes, and plasmids [25]. VIBRANT [26] relies on a “v-score” metric—computed by aligning sequences against hidden Markov model (HMM) protein profiles in public databases—and uses a neural network to distinguish among prokaryotic double-stranded DNA viruses, single-stranded DNA viruses, and RNA viruses. The latest version, VirSorter2 [27], adopts a multi-classifier architecture that substantially improves the detection of underrepresented viral groups, including RNA viruses, giant viruses, and bacteriophages.

Beyond viruses, plasmids represent another class of MGEs that are pervasive in environmental microbiomes, attracting particular attention in host-associated microenvironments and wastewater treatment systems. Plasmids can confer novel adaptive traits upon their hosts through inter-host transfer or gene exchange with chromosomes [28]. For instance, they frequently carry genes associated with resource metabolism, antibiotic resistance, heavy metal tolerance, and defense mechanisms, thereby enhancing the genetic plasticity and survival advantage of their hosts in fluctuating environments. Currently, PPR-Meta [25] and geNomad [29] are capable of simultaneously identifying both viral and plasmid sequences in a single run. Among them, geNomad integrates gene-feature-based and deep learning approaches, providing a unified framework for the classification and annotation of MGEs. In addition, several tools are specifically designed for plasmid identification, including cBar [30], PlasFlow [31], PlaScope [32], and PlasClass [33]. PlaScope relies on species-specific databases for similarity searches, whereas the remaining tools employ various machine learning algorithms operating on k-mer frequencies to perform plasmid prediction.

In contrast, classification methods targeting eukaryotic contigs remain relatively underdeveloped in metagenomics, despite the widespread presence of microbial eukaryotes across diverse ecosystems, including host-associated habitats [34], deep-sea sediments [35], and geothermal springs [36]. Although amplicon sequencing has been successfully employed to reveal eukaryotic diversity [37,38], and genomic and transcriptomic analyses have progressively uncovered their metabolic potential [39–42], our overall understanding of the genomes of diverse microbial eukaryotes remains limited [43]. Alignment-based tools such as Kaiju [44] and MetaEuk [45] assign taxonomic labels by searching for close matches within reference databases; however, their performance is constrained by the coverage and completeness of existing eukaryotic reference genomes [41]. To overcome this limitation, alignment-free identification methods have also been developed. For example, EukRep [46] pioneered the use of k-mer frequencies combined with a linear support vector machine (SVM) to classify metagenomic contigs as either eukaryotic or prokaryotic in origin; Tiara [47] introduced a deep learning architecture for eukaryotic sequence recognition; and Whokaryote [48] employs a random forest model to distinguish prokaryotic from eukaryotic sequences based on gene-structure features.

In metagenomics, although prokaryotic sequences are relatively tractable for classification, the precise delineation of viral, plasmid, and eukaryotic sequences remains challenging. Unified multi-class classification has previously been explored by DeepMicroClass [49], which predicts prokaryotes, eukaryotes, plasmids, and host-associated viral groups using deep neural models, and by 4CAC [50], which performs four-way classification of prokaryotes, viruses, plasmids, and microeukaryotes using length-adjusted XGBoost models followed by assembly-graph refinement. CamFi is therefore not presented as the first unified four-class classifier. Its contribution is a sequence-based architecture that integrates overlapping nucleotide-triplet patterns with multi-scale feature extraction and attention-based fusion to maintain stable classification across contig lengths.

## Datasets and methods

### Datasets

We collected four types of sequences: prokaryotic chromosomal sequences, eukaryotic chromosomal sequences, plasmid sequences and viral sequences.

For prokaryotic chromosome sequences, we downloaded all prokaryotic genomes with the assembly status labeled as “Complete Genome” from the NCBI Genome database on September 20, 2025. This dataset encompasses representative species from both the domain Bacteria and the domain Archaea, ensuring comprehensive coverage of the major branches of the prokaryotic phylogenetic tree.

For eukaryotic chromosome sequences, this data component was constructed by integrating multiple authoritative databases. First, following the eukaryotic taxonomic framework adopted by Kaiju [44] and the PR2 database [51], we extracted genome or gene sequences belonging to 612 taxonomic units. These taxonomic units cover the major protist supergroups, including Amoebozoa, Apusozoa, Cryptophyceae, Euglenozoa, Stramenopiles, Alveolata, Rhizaria, Haptista, Heterolobosea, Metamonada, Rhodophyta, Chlorophyta, and Glaucocystophyceae. To broaden the taxonomic representation of marine microeukaryotes, we also incorporated transcriptomic sequences released by the MMETSP project [41], comprising 32,073,625 high-quality transcripts from 306 marine eukaryotic plankton species and their endosymbionts across more than 40 eukaryotic phyla. MMETSP is a transcriptome resource and therefore primarily represents expressed, spliced sequences; unlike whole-genome shotgun metagenomic contigs, these sequences do not preserve intronic and intergenic regions in their genomic context. We retained MMETSP to improve taxonomic coverage, but evaluations involving this component should not be interpreted as direct evidence of performance on eukaryotic genomic contigs.

For plasmid sequences, we used the PLSDB 2025 update released online in November 2024 [52]. This release contains 72,360 plasmid records collected from RefSeq, GenBank, and the literature. After downloading the updated release, we repeated identifier cleaning and sequence deduplication, regenerated the training, validation, and test partitions, and retrained CamFi with the updated plasmid dataset.

For viral sequences, we extracted bacteriophage and eukaryotic host-associated virus genomes from the Virus–Host DB [53], which contains 5,209 prokaryotic viruses and 12,148 eukaryotic viruses. Additionally, IMG/VR [54] was incorporated as a supplementary database.

Given that plasmid-derived DNA fragments are frequently co-assembled or co-annotated with chromosomal sequences in the NCBI Genome database—a contamination that can lead to erroneous taxonomic assignments or functional annotation biases—we performed cross-referencing of all prokaryotic chromosome sequences against the PLSDB plasmid database. This filtration strategy comprised two layers: (i) removing all entries annotated as “plasmid” from the prokaryotic chromosome dataset, and (ii) screening the sequence identifiers of the prokaryotic chromosomes to exclude any that were also present in PLSDB.

For dataset partitioning, we adopted a stratified group-splitting strategy based on taxonomic hierarchy. All sequences belonging to the same taxonomic group were assigned entirely to one of the training, validation, or test subsets, thereby reducing phylogenetic overlap among splits. Owing to the maturity of bacterial and archaeal taxonomy, sequences from these domains were partitioned at the genus level. MMETSP transcripts were partitioned by sample identifier because some samples have incomplete taxonomic annotations and transcriptome assemblies can contain misassemblies. This sample-level separation prevents direct sample overlap, but it does not eliminate the domain difference between transcript-derived sequences and genomic metagenomic contigs. Viral sequences were randomly partitioned because of their rapid evolution, diverse host ranges, and heterogeneous genome architectures.

All data groups were partitioned at a ratio of 70% training, 15% validation, and 15% test sets. To further prevent test set leakage, we implemented a redundancy-removal filter based on Mash distance: any test set sequence exhibiting a Mash distance less than 0.1 to any sequence in the training or validation set was excluded from the test set.

### Benchmark dataset

To systematically evaluate the classification performance of CamFi across different community composition scenarios for various contig types, we constructed two categories of benchmark datasets: datasets with varying contig lengths and datasets with varying class proportions.

Varying-length contig dataset. We divided the dataset into five groups according to contig length, with thresholds set at 2,000 bp, 3,000 bp, 5,000 bp, 8,000 bp, and 10,000 bp. To eliminate differences in total sequence amount across groups, equal sampling was performed using 120,000 bp—the common multiple of the five length thresholds—as the benchmark, ensuring that each group contained the same total sequence length and that all four taxonomic categories were uniformly represented within each group. This experimental design was intended to verify CamFi’s adaptability to input sequences of arbitrary length and its classification stability under varying length conditions.

Varying class-proportion dataset. This dataset comprises 1,000 contigs, each 10,000 bp in length. We designed two community composition scenarios, with eukaryotic chromosomes and plasmids serving as the dominant component, respectively. Each scenario consists of a mixture of the dominant component and the three other sequence categories. The ratio of the dominant component to the other components was set to 1:9, 3:7, 5:5, 7:3, and 9:1, thereby simulating a gradient from low to high relative abundance.

Additionally, to evaluate the generalization capacity of CamFi under conditions that more closely resemble real metagenomic environments, we employed the CAMI II (Critical Assessment of Metagenome Interpretation II) marine dataset. CAMI II is a widely recognized comprehensive benchmarking resource that provides simulated and real microbial communities spanning various levels of complexity, with the aim of standardizing the performance evaluation of metagenomic analysis tools. In this study, we specifically selected the marine subset containing both prokaryotic and viral sequences to compare the classification accuracy and robustness of CamFi and existing viral sequence classifiers.

For direct comparison of unified multi-class methods, we additionally constructed a balanced four-class benchmark containing 3,624 contigs: 906 prokaryotic chromosome sequences, 906 viral sequences, 906 plasmid sequences, and 906 eukaryotic chromosome sequences.

### Model design

#### 1D-DRCN.

The 1D-DRCN (one-dimensional dilated residual convolution) module serves as the fundamental building block of the network architecture proposed in this study. It is designed to effectively expand the receptive field without substantially increasing model parameters, while preserving the integrity of gradient flow. Centered on one-dimensional dilated convolutions, this module integrates batch normalization, PReLU activation, and residual connections to form an efficient and stable feature extraction unit, as illustrated in Fig 1.

**Fig 1:**
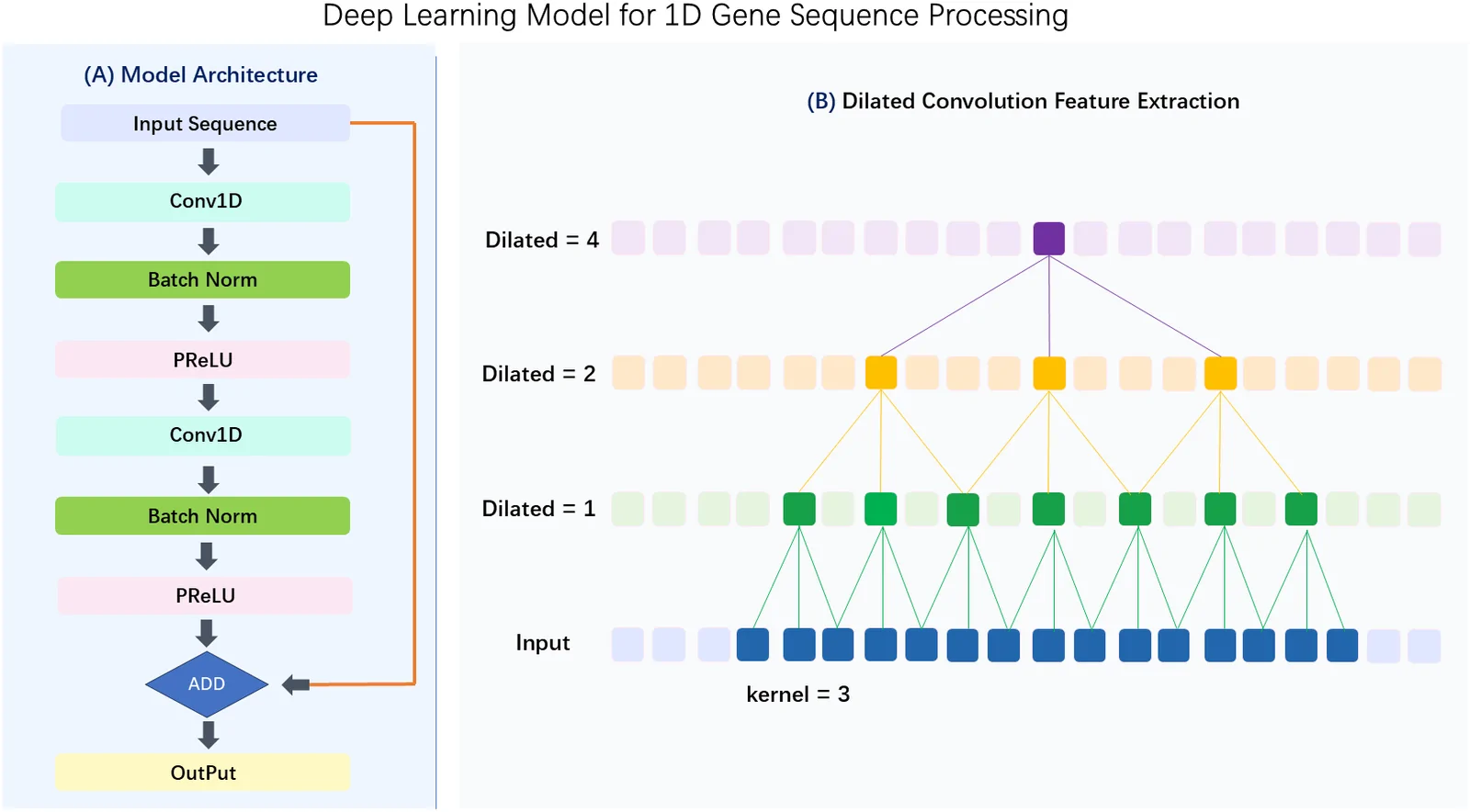
Structure of 1D-DRCN.

Given an input feature $\alpha \in R^{C_{in}}$, ($C_{in}$ denotes the number of input channels and L denotes the sequence length) the forward computation of the 1D-DRCN module can be formally expressed as Equations (1)–(3):


$$\begin{array}{c} h_{\text{1}}=PReLU(BN(Conv\text{1}d_{d}(\text{x}))) \end{array}$$ (1)

$$\begin{array}{c} h_{\text{2}}=PReLU(BN(Conv\text{1}d_{d}(h_{\text{1}}))) \end{array}$$ (2)

$$\begin{array}{c} y=\;h_{\text{2}}+Proj(x) \end{array}$$ (3)

$Conv\text{1}d_{d}$ denotes a one-dimensional convolution with kernel size 3 and dilation rate d; BN denotes the batch normalization layer; PReLU is the parametric ReLU activation function; Proj(x) denotes the projection operation: when the number of input and output channels is identical, an identity mapping is applied; otherwise, a 1*1 convolution is used for channel alignment.

The effective receptive field (RF) of this module is jointly determined by the dilation rate d and the convolution kernel size. For a single DRCN block, the overall receptive field is given by Equation (4):


$$\begin{array}{c} RF=(k-\text{1})\cdot d+\text{1}=\text{2}d+\text{1}\;(k=\text{3}) \end{array}$$ (4)

When multiple DRCN blocks are stacked with dilation rates d_1_, d_2_,…,d_n_, the total receptive field accumulates linearly, as expressed in Equation (5):


$$\begin{array}{c} {RF}_{total}=\text{1}+\sum_{i=\text{1}}^{n}\text{2}d_{i}. \end{array}$$ (5)

This property enables the model to capture long-range dependencies at a remarkably low parameter cost.

#### Multi-scale architecture.

Building upon the 1D-DRCN module described above, we propose CamFi, a network designed to perform accurate classification of input nucleotide sequences into four categories: prokaryotic chromosomes, eukaryotic chromosomes, plasmids, and viruses. The network consists of three parallel pathways that model sequence features at long, medium, and short scales, respectively, in order to capture sequence patterns corresponding to distinct biological functional units.

First, the input sequence is encoded as a one-hot matrix: nucleotides A, C, G, and T are mapped to the four-dimensional one-hot vectors [1,0,0,0], [0,1,0,0], [0,0,1,0], and [0,0,0,1], respectively, while any non-standard bases are uniformly encoded as [0,0,0,0].

To enhance the model’s awareness of double-stranded DNA symmetry, we simultaneously generate the reverse complementary strand of the input sequence: the one-hot matrix is reversed in row order, and the positions of A ↔ T and C ↔ G within each row are swapped, thereby yielding the one-hot representation of the complementary strand.

The specific design of each scale-specific pathway is illustrated in Fig 2.

**Fig 2:**
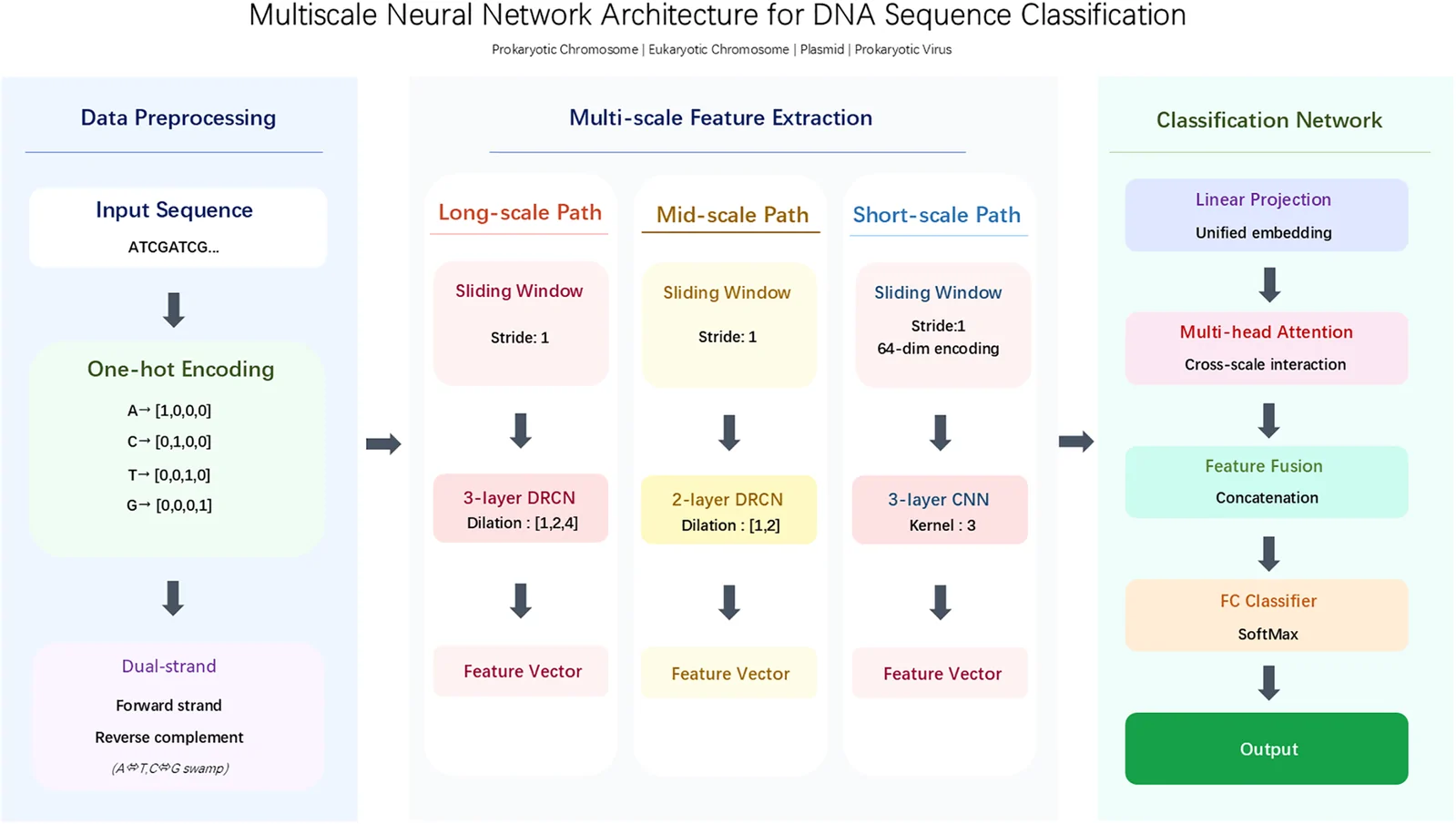
Structure of CamFi.

Long-scale pathway: This pathway consists of three stacked 1D-DRCN modules with dilation rates of 1, 2, and 4, yielding a receptive field of 15 bp. At this scale, the model is able to recognize biologically important signals such as ribosome binding sites and transcription factor binding motifs.

Medium-scale pathway: This pathway consists of two stacked 1D-DRCN modules with dilation rates of 1 and 2, yielding a receptive field of 7 bp. This pathway captures short local nucleotide patterns at an intermediate scale, complementing the broader context modeled by the long-scale pathway and the triplet patterns modeled by the short-scale pathway.

Short-scale pathway: The sequence is scanned using an overlapping 3-bp window with a stride of 1. Each resulting 3-mer is mapped to a 64-dimensional one-hot vector representing one of the 64 possible nucleotide triplets and is then processed by a three-layer CNN block. Because these windows overlap and no open reading frame or reading frame is inferred, the extracted features represent codon-like triplet composition rather than synonymous codon usage bias. This pathway is intended to learn discriminative local triplet patterns directly from raw nucleotide sequences.

Finally, the fused features are fed into a multi-head attention mechanism, which dynamically models the interactions among features extracted at different scales. The weighted features produced by the attention mechanism are concatenated and passed to a fully connected classifier, enabling end-to-end prediction of the four biological origins.

### Model training

All experiments in this study were conducted on an RTX 4090 platform. Hyperparameter selection used a 3 x 3 grid comprising learning rates of 0.001, 0.0001, and 0.00001 and batch sizes of 64, 128, and 192 (Fig 3). The best validation F1 score was 0.9415 at a learning rate of 0.0001 and a batch size of 128. At this learning rate, the validation F1 scores were 0.9248, 0.9415, and 0.9321 for batch sizes 64, 128, and 192, respectively. A learning rate of 0.001 produced F1 scores from 0.9127 to 0.9180, whereas 0.00001 was less stable, ranging from 0.8571 to 0.9192. We therefore selected a learning rate of 0.0001 and a batch size of 128 for subsequent experiments. Complete grid-search results are provided in S1 Table.

**Fig 3:**
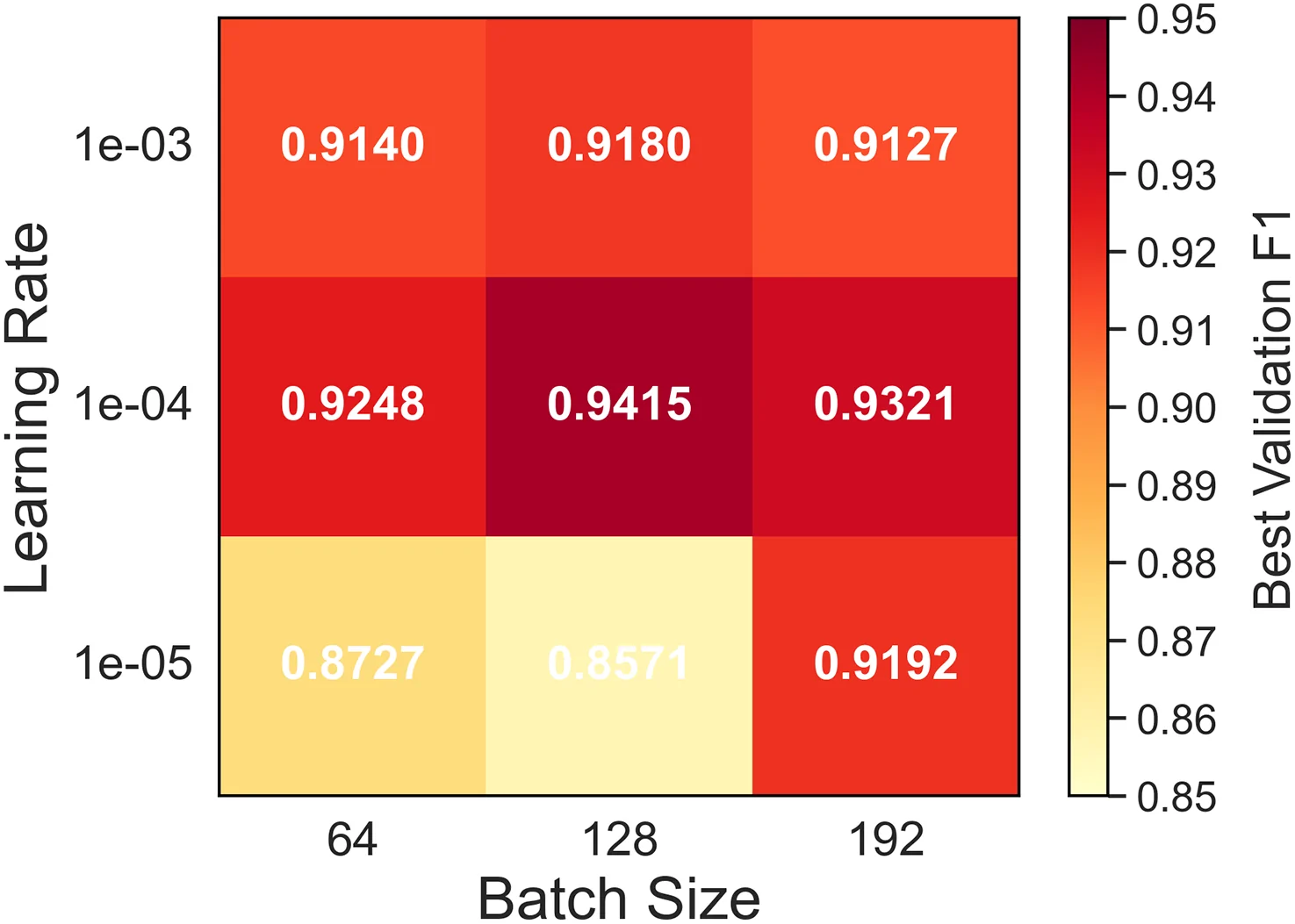
Sensitivity analysis of hyperparameters on the validation set F1 score: learning rate and batch size.

Across all configurations, the performance metrics were observed to plateau after 100 training epochs. The best-performing configuration, as determined by the evaluation metrics, is presented in Table 1:

**Table 1:** Hyperparameter config.

Hyperparameters	Value
Epoch	100
Batch size	128
Learning Rate	0.0001

Given that the total data volume reaches 1TB which far exceeds GPU memory capacity, we designed a multi-scale dynamic sampling strategy that collaboratively optimizes the training process at three hierarchical levels.

First, at the epoch level, a deterministic seeding mechanism was introduced, whereby each training epoch randomly samples from different genomic regions.

Second, at the fragment-scale level, each sample was randomly truncated to one of six target lengths ranging from 500 to 8,000 bp, in order to capture genomic features at different resolutions. For sequence ends shorter than the window length, zero-vector padding was applied to maintain uniform input dimensions. For the pooling of zero-padded regions, we adopted masked average pooling—that is, averaging was performed exclusively over the unpadded portion of the sequence.

Finally, at the positional level, the starting site of each fragment of a given length was randomly cropped within the valid range of its parent sequence, ensuring that the subsequences actually fed into the model remain distinct even when the sampling schedule is repeated.

## Results and analysis

In this section, we systematically evaluate the classification performance of CamFi across various contig types under different community composition scenarios, encompassing varying contig lengths, varying class proportions, and the CAMI II dataset. Detailed descriptions of all benchmark datasets are provided in Section 2.3.

We adopted precision, recall, and F1 score as the evaluation metrics for model performance. Precision is defined as the proportion of correctly predicted positive samples among all samples predicted as positive; recall is defined as the proportion of correctly predicted positive samples among all actual positive samples. The F1 score is the harmonic mean of precision and recall, as expressed in Equation (6):


$$\begin{array}{c} F_{\text{1}}=\text{2}\times \frac{Precision\times Recall}{Precision+Recall} \end{array}$$ (6)

### Varying-length contig dataset

Existing metagenomic classification methods often rely on data preprocessing and are highly sensitive to input contig length, which severely limits the robustness of classification systems in practical applications. To evaluate the stability of CamFi as a multi-class predictor across different sequence lengths, we systematically tested it on five contig datasets with varying length thresholds (2,000 bp to 10,000 bp), using the macro-averaged F1 score as the primary evaluation metric. Each scatter point represents a single experimental run; six replicate runs were performed to exclude the influence of random seed effects.

Across six random seeds, the mean macro-F1 scores at the 2,000, 3,000, 5,000, 8,000, and 10,000 bp thresholds were 93.22%, 92.03%, 94.97%, 96.96%, and 95.80%, respectively (Fig 4). The corresponding sample SDs were 1.45%, 1.52%, 2.04%, 1.23%, and 4.58%. Performance was therefore not strictly monotonic: the highest mean occurred at 8,000 bp, followed by a small decrease at 10,000 bp. Nevertheless, the 10,000 bp mean exceeded the 2,000 bp mean by 2.59 percentage points, a relative increase of 2.77%. The greater variation at 10,000 bp is consistent with the smaller number of valid sequences available at the longest threshold. Per-seed precision, recall, and F1 values are provided in S2 Table.

**Fig 4:**
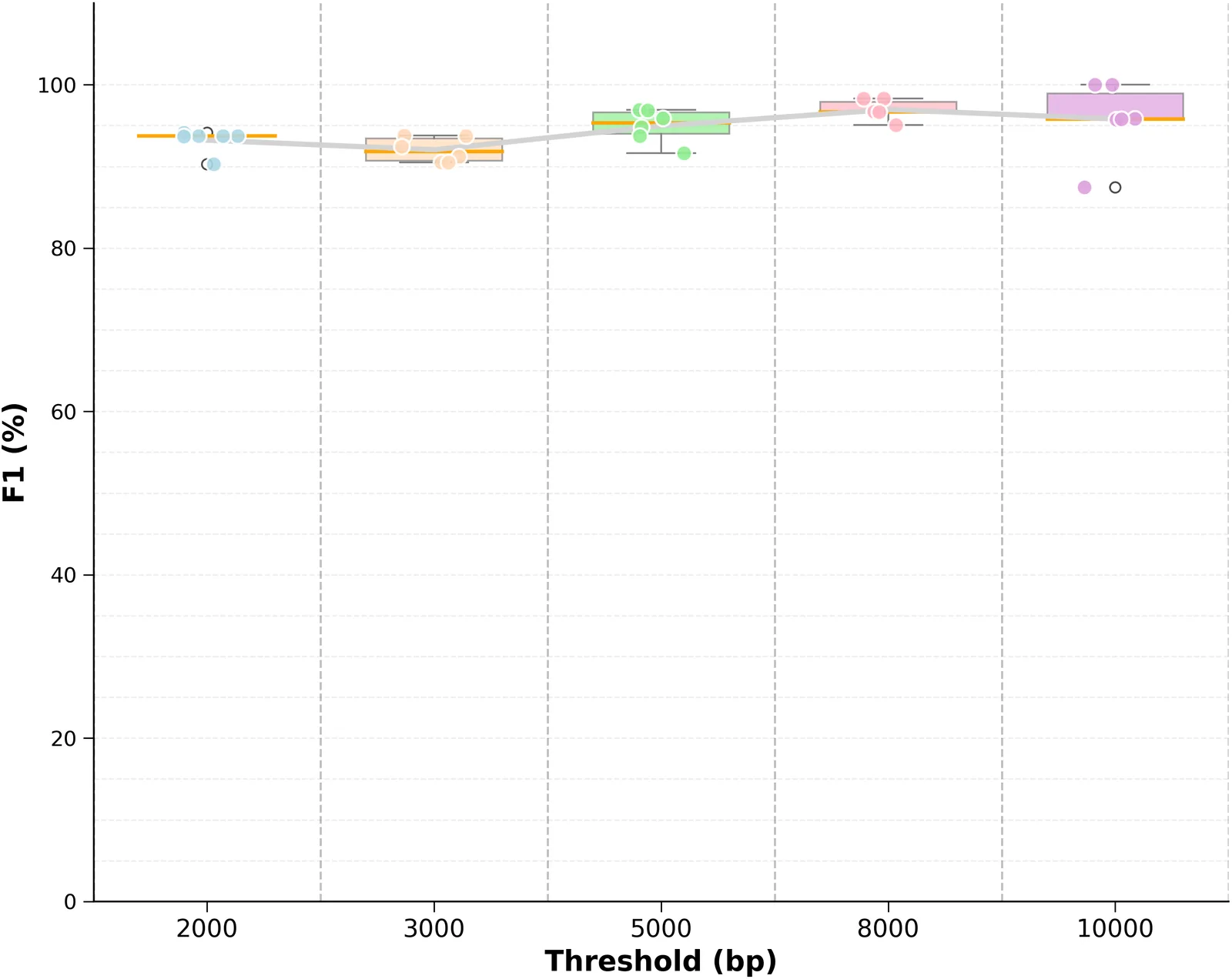
The effect of sequence length filtering thresholds on classification performance.

### Varying class-proportion dataset

To assess the model’s performance under different community structures, we conducted a stratified analysis of the dataset based on the dominant component class. The results demonstrate that, as a multi-class prediction framework, CamFi not only achieves excellent overall classification performance but also exhibits strong discriminative power in decomposed binary classification subtasks, surpassing existing state-of-the-art methods in the majority of the comparison scenarios.

#### Eukaryotic chromosome.

We compared CamFi with Whokaryote and TIARA for eukaryotic chromosome identification using recall and F1 score across five class-proportion settings. CamFi exceeded Whokaryote in every setting and matched or exceeded TIARA, including equal recall and F1 values in the 9:1 setting. Fig 5 displays the individual setting values, overall one-way ANOVA p-values, and pairwise Welch’s t-test p-values. Per-setting metrics are provided in S3 Table.

**Fig 5:**
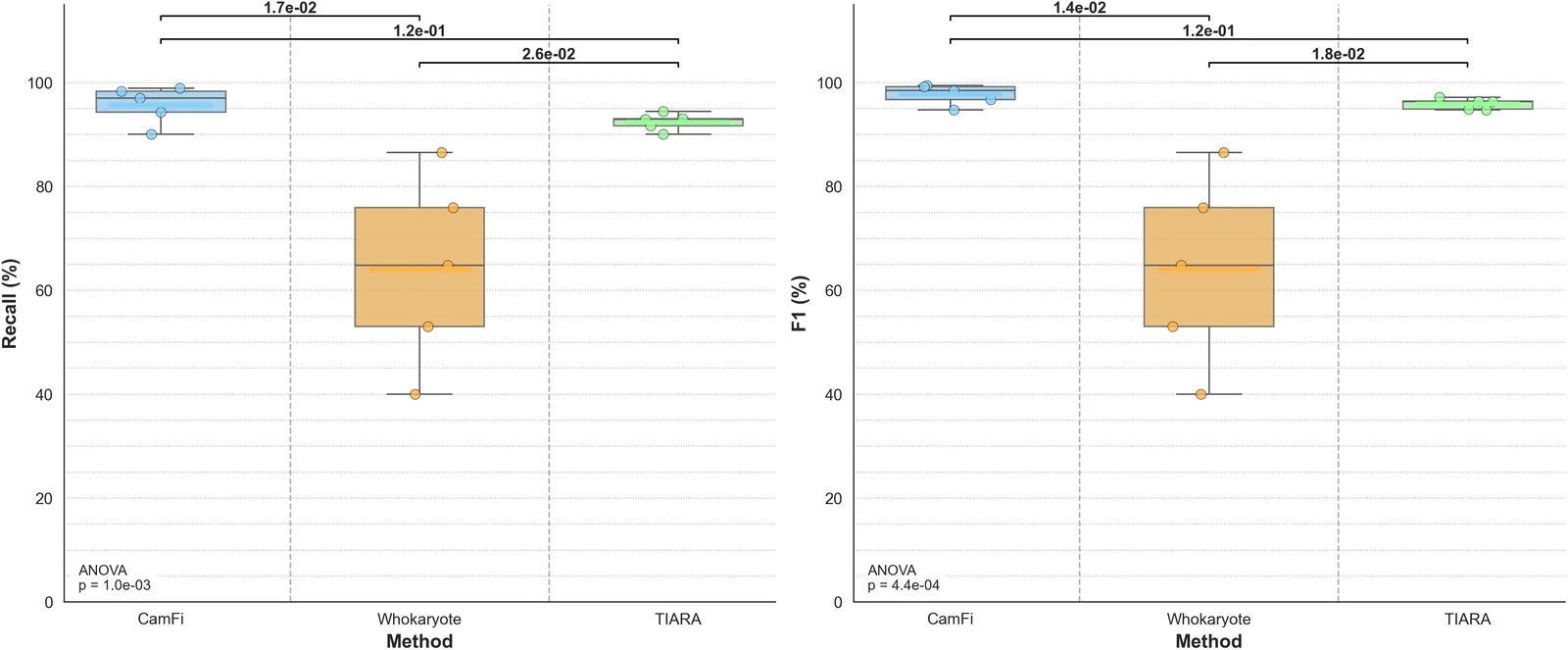
Comparison of the performance of three methods for classifying eukaryotic sequence fragments.

Across the five settings, CamFi achieved a mean recall of 95.70%, compared with 92.39% for TIARA and 64.04% for Whokaryote. The corresponding mean F1 scores were 97.70%, 95.88%, and 64.04%, respectively. CamFi therefore had the highest mean recall and F1, while Whokaryote showed substantially greater variation across the class-proportion settings.

One-way ANOVA indicated overall differences among the three methods for both recall and F1 score. Pairwise Welch’s t-tests supported differences between CamFi and Whokaryote for recall and F1. The smaller differences between CamFi and TIARA were not statistically significant for either recall or F1. These tests summarize variation across the five class-proportion settings.

The Whokaryote result requires cautious interpretation because the eukaryotic evaluation component includes MMETSP transcript assemblies. Whokaryote uses gene-structure features, including intergenic distances, that are informative for genomic contigs but are largely absent from spliced transcript sequences. Its performance on the transcript-derived subsets therefore does not represent its expected performance on whole-genome metagenomic contigs; the comparison is reported only as a within-dataset descriptive benchmark.

#### Plasmids.

We compared the performance of CamFi with two methods, geNomad and PlasFlow, on the plasmid identification task. The results are presented in Fig 6.

**Fig 6:**
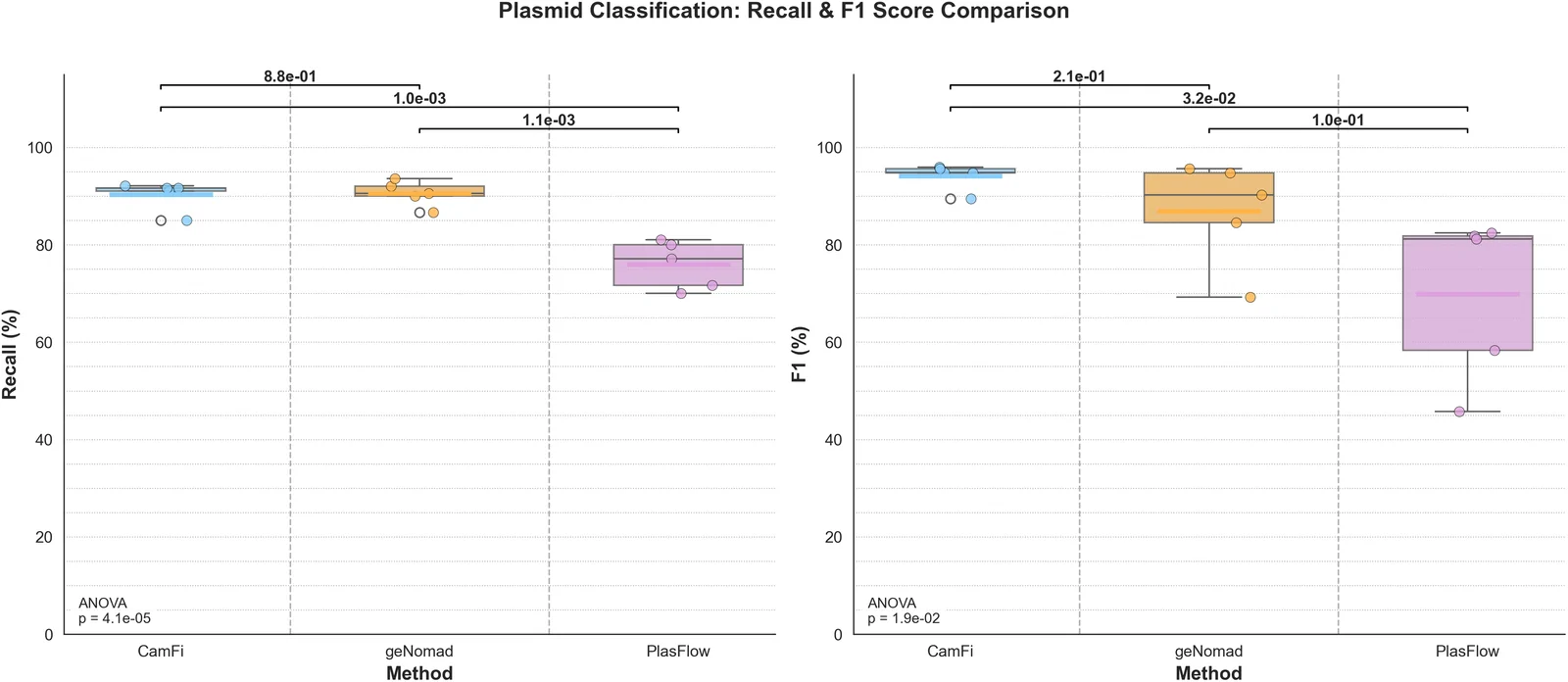
Comparison of recall and F1 score for plasmid sequence classification.

Across the five class-proportion settings, geNomad achieved a mean recall of 90.56%, CamFi achieved 90.30%, and PlasFlow achieved 75.96%. The overall recall difference was supported by one-way ANOVA. Pairwise Welch’s t-tests yielded p = 0.885 for CamFi versus geNomad and p = 1.04e-03 for CamFi versus PlasFlow. For F1 score, CamFi achieved the highest mean of 94.13%, compared with 86.87% for geNomad and 69.90% for PlasFlow. The overall F1 difference yielded p = 0.0186 by one-way ANOVA; pairwise tests yielded p = 0.211 for CamFi versus geNomad and p = 0.0319 for CamFi versus PlasFlow. Per-setting precision, recall, and F1 values are provided in S4 Table.

Classification became more difficult as the proportion of plasmids decreased. When non-plasmid sequences accounted for 90% of the benchmark, CamFi retained an F1 score of 89.47%, whereas PlasFlow declined to 45.71%, a difference of 43.76 percentage points. These results indicate that CamFi was less sensitive to class-composition changes in the present benchmark; however, the statistical results above are based on five composition settings and should not be interpreted as evidence of universal superiority across independent datasets.

### CAMI II

Additionally, we employed the CAMI II marine dataset to compare the performance of CamFi, VIBRANT, PPR-Meta, DeepVirFinder, VirSorter2, and geNomad on the virus classification task, as shown in Fig 7.

**Fig 7:**
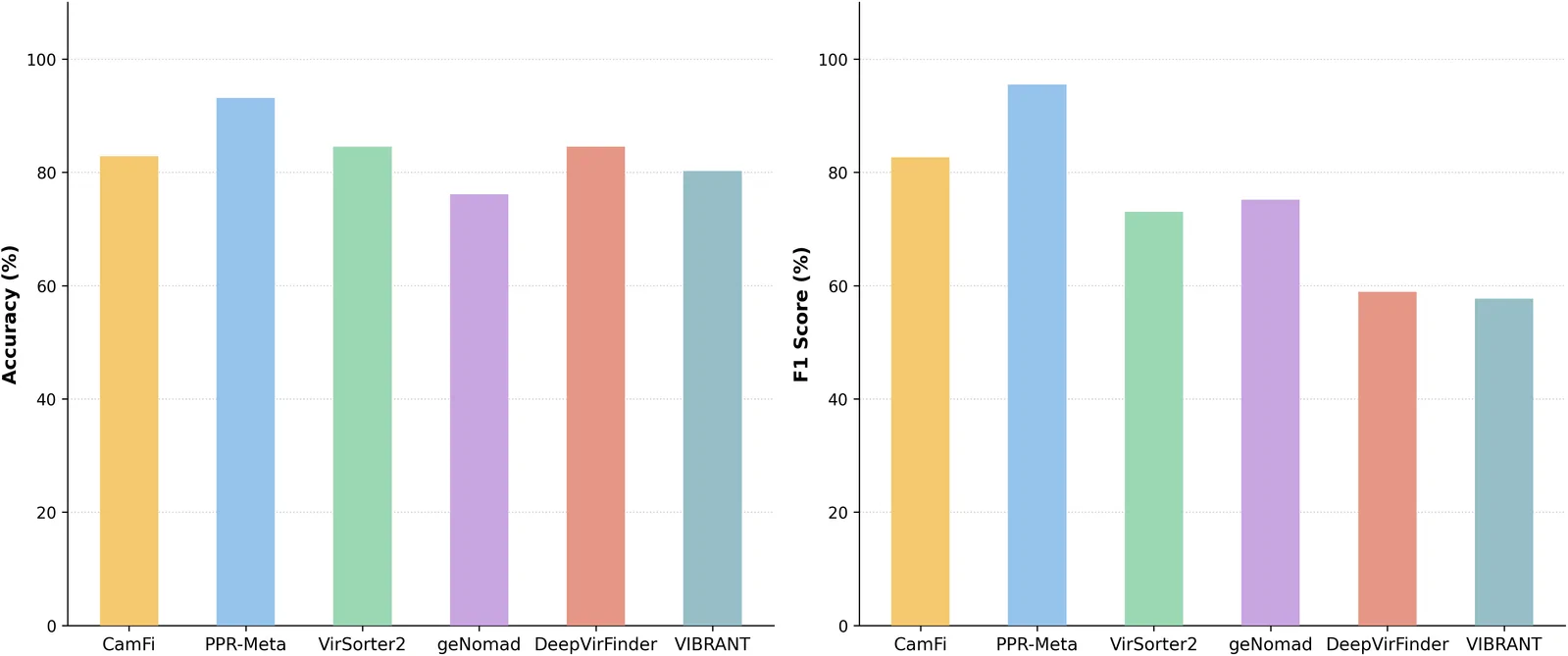
Aggregate accuracy and F1 score of six virus classification methods on the CAMI II dataset.

On the CAMI II marine dataset, CamFi achieved a weighted F1 of 82.65%, ranking second among six methods based on reported aggregate metrics. Because only one aggregate result was available for each method, no valid cross-method significance test could be performed; the ranking is therefore descriptive. Aggregate method results and CamFi sample-level metrics are provided in S5 and S6 Tables, respectively.

### Four-class benchmark comparison

To compare CamFi with existing unified classifiers under an identical four-class setting, we evaluated CamFi, DeepMicroClass, and the sequence-based XGBoost stage of 4CAC (4CAC-XGBoost) on the balanced benchmark described above. Macro-averaged F1 was used as the common evaluation metric, as shown in Fig 8.

**Fig 8:**
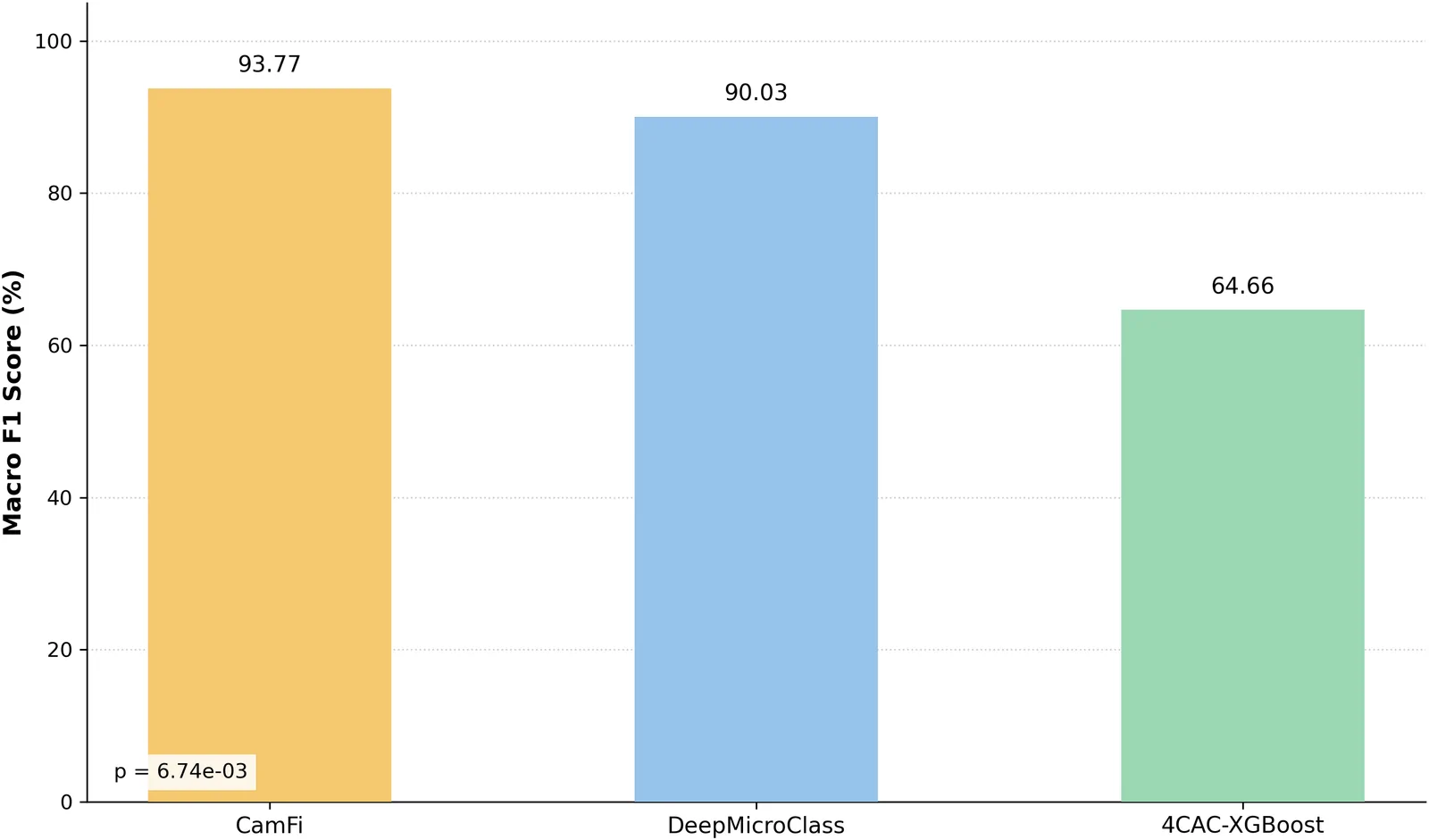
Macro-F1 comparison of CamFi, DeepMicroClass, and 4CAC-XGBoost on the balanced four-class benchmark.

CamFi achieved the highest macro-F1 score of 93.77%, followed by DeepMicroClass at 90.03% and 4CAC-XGBoost at 64.66%. The overall comparison across the five matched contig-length strata yielded p = 6.74e-03. Thus, CamFi exceeded DeepMicroClass by 3.74 percentage points and 4CAC-XGBoost by 29.11 percentage points on this benchmark. The overall and length-stratified results are provided in S7 and S8 Tables, respectively.

## Discussion

Across the evaluated benchmarks, CamFi combined strong general four-class performance with task-dependent differences relative to specialized methods. On the balanced four-class benchmark, CamFi achieved a macro-F1 of 93.77%, compared with 90.03% for DeepMicroClass and 64.66% for the sequence-based XGBoost stage of 4CAC. In contrast, on the CAMI II virus benchmark, CamFi ranked second by aggregate F1 and remained below PPR-Meta. These results position CamFi as an extension of existing unified classification approaches rather than the first method of its kind. They support its utility under the present benchmark conditions without implying universal superiority across datasets or task-specific classifiers.

The observed performance is consistent with the complementary sequence representations learned by the three pathways. The long- and medium-scale branches model local sequence context at different receptive-field widths, whereas the short-scale branch represents overlapping nucleotide triplets. This short-scale representation differs from the ORF-based codon analysis used by PPR-Meta [25], which predicts open reading frames and then measures synonymous codon frequencies. CamFi instead maps every overlapping 3-mer to one of 64 triplet categories without inferring a reading frame. Its features should therefore be interpreted as codon-like triplet composition rather than synonymous codon usage bias. Avoiding explicit gene prediction may be beneficial for fragmented or partially coding contigs, although it also removes reading-frame-specific information.

The multi-scale design nevertheless operates within a finite sequence context. Three stacked 1D-DRCN modules with dilation rates of 1, 2, and 4 give the long-scale pathway an effective receptive field of approximately 15 bp. This context can represent short local patterns at the scale of ribosome-binding sites or transcription-factor motifs, but it cannot span complete promoter regions, which generally exceed 30 bp in prokaryotes and may extend for hundreds of base pairs in eukaryotes. Increasing the number and dilation rates of convolutional layers could broaden the accessible context in future versions while limiting growth in the number of model parameters.

The scope of the eukaryotic results is also constrained by the composition of the training and evaluation data. MMETSP contributes expressed, exon-rich transcript sequences, whereas environmental whole-genome shotgun contigs may contain introns, intergenic regions, repeats, and regulatory elements. Consequently, evaluation on held-out MMETSP samples measures generalization within the transcriptomic domain rather than direct generalization to eukaryotic metagenomic DNA. The Whokaryote comparison is particularly sensitive to this difference because that method uses gene structure and intergenic-distance features that are largely absent from spliced transcripts. The current eukaryotic comparison should therefore be read as a within-dataset benchmark. Evaluation on taxonomically diverse whole-genome assemblies and eukaryote-containing simulated or real metagenomes will be needed to establish broader genomic-domain generalization.

## Conclusion

This study addresses the complexity of genetic element classification in metagenomic analysis by introducing CamFi, a deep learning framework based on codon awareness and multi-scale feature fusion. We have constructed a unified classification system capable of simultaneously identifying four key biological sequence categories: prokaryotic chromosomes, eukaryotic chromosomes, plasmids, and viruses.

Systematic evaluation demonstrates that CamFi achieves strong four-class classification performance under the tested community compositions. It attained the highest mean F1 in the eukaryotic chromosome and plasmid comparisons, although its difference from TIARA in the eukaryotic comparison was not statistically significant. On CAMI II, CamFi ranked second by aggregate F1 behind PPR-Meta and ahead of the other four evaluated virus classifiers. In the balanced four-class benchmark, CamFi achieved 93.77% macro-F1, compared with 90.03% for DeepMicroClass and 64.66% for 4CAC-XGBoost. Together with its stable performance across contig-length thresholds, these results support CamFi as a general-purpose classifier under the present benchmark conditions.

Although CamFi has demonstrated strong performance in the classification of four metagenomic sequence categories, future work will explore the following directions to further refine this framework.

First, given the massive scale of metagenomic data, we will focus on optimizing the computational efficiency of the model by introducing lightweight architectural strategies or more efficient neural network designs.

Second, to overcome the coverage limitations of existing reference databases and to discover more novel sequences, we plan to explore DNA sequence embedding approaches, thereby substantially enhancing the model’s generalization capacity for unknown sequences.

Additionally, future efforts will aim to refine classification granularity—specifically, to achieve precise discrimination among viral types and plasmid subgroups. We will also seek to integrate gene-structural features and protein domain information to construct more accurate hybrid classifiers, equipping the framework to meet the analytical challenges posed by extremely complex environmental samples.

## Supporting information

S1 Table Complete hyperparameter grid-search results and validation metrics. (CSV)

S2 Table Precision, recall, and macro-F1 results across contig-length thresholds and random seeds. (CSV)

S3 Table Eukaryotic chromosome classification metrics across five class-composition settings. (CSV)

S4 Table Plasmid classification metrics across five class-composition settings. (CSV)

S5 Table Aggregate method comparison on the CAMI II marine dataset. (CSV)

S6 Table Sample-level CamFi metrics on the CAMI II marine dataset. (CSV)

S7 Table Macro-F1 comparison of CamFi, DeepMicroClass, and 4CAC-XGBoost on the balanced four-class benchmark. (CSV)

S8 Table Length-stratified macro-F1 results on the balanced four-class benchmark. (CSV)
