# Supplementary material for: Codon-aware multi-scale feature fusion for metagenomic sequence classification
Source: PLoS One. 2026 Oct 5;21(10):e0359090. doi: 10.1371/journal.pone.0359090 (PMC13637925; doi:10.1371/journal.pone.0359090)
Supplement: Supplementary file 1 [file pone.0359090.s009.docx]

**Manuscript ID: PONE-D-26-25179**

**Manuscript Title: Codon-Aware Multi-Scale Feature Fusion for Metagenomic Sequence Classification**

**Authors: Yan Qian, Quanjie Song, Xinyi Xie, and Li Deng**

**Submitted to: PLOS ONE**

**Response to Reviewers' Comments**

Dear Dr. Esposito,

Thank you for the opportunity to revise our manuscript and for the careful and constructive comments from you and the reviewers. We have addressed every comment point by point. Reviewer and editor comments are shown in black italics, our responses and summaries are shown in blue, and the corresponding revised manuscript text is reproduced in green. Relevant revised figures are included where applicable.

Sincerely yours,

Li Deng, PhD
Corresponding author

**Response to the Academic Editor**

***Comment AE1:*** *The manuscript requires deep revision, with particular attention to the pitfalls identified by Reviewer 1.*

Response: Thank you for this overall assessment and for directing our attention to the substantive issues raised by Reviewer 1.

We made the following revisions:

(1) We replaced claims of synonymous codon-usage bias with a precise description of overlapping nucleotide-triplet composition.

(2) We limited the biological interpretation of the 7-bp and 15-bp receptive fields and explicitly discussed the MMETSP transcriptome-to-genome domain gap.

(3) We added direct comparisons with DeepMicroClass and 4CAC-XGBoost, updated the plasmid dataset to PLSDB 2025, repeated the associated experiments, and revised the statistical reporting and conclusions.

**Revised text in the Abstract:**

Microorganisms exert profound influences on both natural ecosystems and human society. As the cornerstone of microbial research, metagenomics relies on the precise analysis of large-scale, multi-source genomic data. However, discriminating among diverse sequence types remains a formidable challenge. To address this, we present CamFi (Codon-aware Multi-scale Feature Fusion), a unified framework integrating overlapping nucleotide-triplet representations with multi-scale dilated convolutions for classifying prokaryotic chromosomes, eukaryotic chromosomes, plasmids, and viruses. Benchmark evaluations demonstrate strong mean F1 scores of 97.70% for eukaryotic chromosomes and 94.13% for plasmids. On the CAMI II marine dataset, CamFi achieved a weighted F1 of 82.65%, ranking second among six methods based on reported aggregate metrics. In a balanced four-class benchmark, CamFi attained a macro-F1 of 93.77%, compared with DeepMicroClass (90.03%) and the XGBoost stage of 4CAC (64.66%). These results establish CamFi as a competitive approach for unified metagenomic contig classification under the evaluated conditions.

**Response to Reviewer #1**

***Comment 1:*** *The overlapping 3-bp sliding-window pathway does not analyze synonymous codon usage bias because it does not infer ORFs or a reading frame.*

Response: Thank you for identifying this important terminology and methodological issue.

We made the following revisions:

(1) We removed statements that CamFi measures synonymous codon-usage bias and now describe the short-scale input as overlapping 3-mers with stride 1.

(2) We explicitly state that the model does not infer an ORF or reading frame and contrast this representation with the ORF-based codon analysis used by PPR-Meta.

**Revised text in the Methods, Short-scale pathway:**

Short-scale pathway: The sequence is scanned using an overlapping 3-bp window with a stride of 1. Each resulting 3-mer is mapped to a 64-dimensional one-hot vector representing one of the 64 possible nucleotide triplets and is then processed by a three-layer CNN block. Because these windows overlap and no open reading frame or reading frame is inferred, the extracted features represent codon-like triplet composition rather than synonymous codon usage bias. This pathway is intended to learn discriminative local triplet patterns directly from raw nucleotide sequences.

**Revised text in the Discussion:**

The observed performance is consistent with the complementary sequence representations learned by the three pathways. The long- and medium-scale branches model local sequence context at different receptive-field widths, whereas the short-scale branch represents overlapping nucleotide triplets. This short-scale representation differs from the ORF-based codon analysis used by PPR-Meta [25], which predicts open reading frames and then measures synonymous codon frequencies. CamFi instead maps every overlapping 3-mer to one of 64 triplet categories without inferring a reading frame. Its features should therefore be interpreted as codon-like triplet composition rather than synonymous codon usage bias. Avoiding explicit gene prediction may be beneficial for fragmented or partially coding contigs, although it also removes reading-frame-specific information.

Fig. 2. Structure of CamFi, including the overlapping triplet pathway

***Comment 2:*** *A 15-bp receptive field is not sufficient to recognize complete promoter regions.*

Response: Thank you for pointing out that our original biological interpretation exceeded the actual receptive field of the model.

We made the following revisions:

(1) We deleted the claim that the 15-bp pathway recognizes complete promoters and removed the corresponding core-promoter claim for the 7-bp pathway.

(2) We now restrict the interpretation to short local patterns and explicitly acknowledge that complete promoters require broader sequence context.

**Revised text in the Methods, Long- and Medium-scale pathways:**

Long-scale pathway: This pathway consists of three stacked 1D-DRCN modules with dilation rates of 1, 2, and 4, yielding a receptive field of 15 bp. At this scale, the model is able to recognize biologically important signals such as ribosome binding sites and transcription factor binding motifs.

Medium-scale pathway: This pathway consists of two stacked 1D-DRCN modules with dilation rates of 1 and 2, yielding a receptive field of 7 bp. This pathway captures short local nucleotide patterns at an intermediate scale, complementing the broader context modeled by the long-scale pathway and the triplet patterns modeled by the short-scale pathway.

**Revised text in the Discussion:**

The multi-scale design nevertheless operates within a finite sequence context. Three stacked 1D-DRCN modules with dilation rates of 1, 2, and 4 give the long-scale pathway an effective receptive field of approximately 15 bp. This context can represent short local patterns at the scale of ribosome-binding sites or transcription-factor motifs, but it cannot span complete promoter regions, which generally exceed 30 bp in prokaryotes and may extend for hundreds of base pairs in eukaryotes. Increasing the number and dilation rates of convolutional layers could broaden the accessible context in future versions while limiting growth in the number of model parameters.

Fig. 2. Structure of CamFi and its three scale-specific pathways

***Comment 3:*** *MMETSP transcriptomes differ from whole-genome metagenomic DNA, and this domain difference can disadvantage Whokaryote.*

Response: Thank you for highlighting the transcriptome-to-genome domain gap and its implications for the Whokaryote comparison.

We made the following revisions:

(1) We now identify MMETSP explicitly as expressed, spliced transcript data and explain why it was retained for taxonomic coverage.

(2) We state that sample-level separation prevents direct sample overlap but does not eliminate the domain gap.

(3) We restrict the Whokaryote result to a within-dataset descriptive comparison and identify genomic-metagenome evaluation as necessary future work.

**Revised text in the Datasets section:**

For eukaryotic chromosome sequences, this data component was constructed by integrating multiple authoritative databases. First, following the eukaryotic taxonomic framework adopted by Kaiju [44] and the PR2 database [49], we extracted genome or gene sequences belonging to 612 taxonomic units. These taxonomic units cover the major protist supergroups, including Amoebozoa, Apusozoa, Cryptophyceae, Euglenozoa, Stramenopiles, Alveolata, Rhizaria, Haptista, Heterolobosea, Metamonada, Rhodophyta, Chlorophyta, and Glaucocystophyceae. To broaden the taxonomic representation of marine microeukaryotes, we also incorporated transcriptomic sequences released by the MMETSP project [41], comprising 32,073,625 high-quality transcripts from 306 marine eukaryotic plankton species and their endosymbionts across more than 40 eukaryotic phyla. MMETSP is a transcriptome resource and therefore primarily represents expressed, spliced sequences; unlike whole-genome shotgun metagenomic contigs, these sequences do not preserve intronic and intergenic regions in their genomic context. We retained MMETSP to improve taxonomic coverage, but evaluations involving this component should not be interpreted as direct evidence of performance on eukaryotic genomic contigs.

For dataset partitioning, we adopted a stratified group-splitting strategy based on taxonomic hierarchy. All sequences belonging to the same taxonomic group were assigned entirely to one of the training, validation, or test subsets, thereby reducing phylogenetic overlap among splits. Owing to the maturity of bacterial and archaeal taxonomy, sequences from these domains were partitioned at the genus level. MMETSP transcripts were partitioned by sample identifier because some samples have incomplete taxonomic annotations and transcriptome assemblies can contain misassemblies. This sample-level separation prevents direct sample overlap, but it does not eliminate the domain difference between transcript-derived sequences and genomic metagenomic contigs. Viral sequences were randomly partitioned because of their rapid evolution, diverse host ranges, and heterogeneous genome architectures.

**Revised text in the Results:**

The Whokaryote result requires cautious interpretation because the eukaryotic evaluation component includes MMETSP transcript assemblies. Whokaryote uses gene-structure features, including intergenic distances, that are informative for genomic contigs but are largely absent from spliced transcript sequences. Its performance on the transcript-derived subsets therefore does not represent its expected performance on whole-genome metagenomic contigs; the comparison is reported only as a within-dataset descriptive benchmark.

**Revised text in the Discussion:**

The scope of the eukaryotic results is also constrained by the composition of the training and evaluation data. MMETSP contributes expressed, exon-rich transcript sequences, whereas environmental whole-genome shotgun contigs may contain introns, intergenic regions, repeats, and regulatory elements. Consequently, evaluation on held-out MMETSP samples measures generalization within the transcriptomic domain rather than direct generalization to eukaryotic metagenomic DNA. The Whokaryote comparison is particularly sensitive to this difference because that method uses gene structure and intergenic-distance features that are largely absent from spliced transcripts. The current eukaryotic comparison should therefore be read as a within-dataset benchmark. Evaluation on taxonomically diverse whole-genome assemblies and eukaryote-containing simulated or real metagenomes will be needed to establish broader genomic-domain generalization.

Fig. 5. Eukaryotic sequence-fragment comparison

***Comment 4:*** *The manuscript should not claim that no unified four-class method exists and should compare CamFi with DeepMicroClass and 4CAC.*

Response: Thank you for correcting our original novelty claim and for recommending the two directly relevant unified classifiers.

We made the following revisions:

(1) We removed the claim that CamFi is the first unified four-class method and added DeepMicroClass and 4CAC to the Introduction and references.

(2) We constructed a balanced benchmark of 3,624 contigs and evaluated CamFi, DeepMicroClass, and the sequence-based XGBoost stage of 4CAC on the identical sequences.

(3) We revised the Discussion to position CamFi as an extension of existing unified approaches rather than the first such classifier.

**Revised text in the Introduction:**

In metagenomics, although prokaryotic sequences are relatively tractable for classification, the precise delineation of viral, plasmid, and eukaryotic sequences remains challenging. Unified multi-class classification has previously been explored by DeepMicroClass [53], which predicts prokaryotes, eukaryotes, plasmids, and host-associated viral groups using deep neural models, and by 4CAC [54], which performs four-way classification of prokaryotes, viruses, plasmids, and microeukaryotes using length-adjusted XGBoost models followed by assembly-graph refinement. CamFi is therefore not presented as the first unified four-class classifier. Its contribution is a sequence-based architecture that integrates overlapping nucleotide-triplet patterns with multi-scale feature extraction and attention-based fusion to maintain stable classification across contig lengths.

**Revised text in the Benchmark Dataset and Results:**

For direct comparison of unified multi-class methods, we additionally constructed a balanced four-class benchmark containing 3,624 contigs: 906 prokaryotic chromosome sequences, 906 viral sequences, 906 plasmid sequences, and 906 eukaryotic chromosome sequences.

To compare CamFi with existing unified classifiers under an identical four-class setting, we evaluated CamFi, DeepMicroClass, and the sequence-based XGBoost stage of 4CAC (4CAC-XGBoost) on the balanced benchmark described above. Macro-averaged F1 was used as the common evaluation metric, as shown in Fig. 8.

CamFi achieved the highest macro-F1 score of 93.77%, followed by DeepMicroClass at 90.03% and 4CAC-XGBoost at 64.66%. The overall comparison across the five matched contig-length strata yielded p = 6.74e-03. Thus, CamFi exceeded DeepMicroClass by 3.74 percentage points and 4CAC-XGBoost by 29.11 percentage points on this benchmark.

**Revised text in the Discussion:**

Across the evaluated benchmarks, CamFi combined strong general four-class performance with task-dependent differences relative to specialized methods. On the balanced four-class benchmark, CamFi achieved a macro-F1 of 93.77%, compared with 90.03% for DeepMicroClass and 64.66% for the sequence-based XGBoost stage of 4CAC. In contrast, on the CAMI II virus benchmark, CamFi ranked second by aggregate F1 and remained below PPR-Meta. These results position CamFi as an extension of existing unified classification approaches rather than the first method of its kind. They support its utility under the present benchmark conditions without implying universal superiority across datasets or task-specific classifiers.

Fig. 8. Macro-F1 comparison on the balanced four-class benchmark

***Comment 5:*** *The 2021 PLSDB release is outdated and should be replaced with the newer release.*

Response: Thank you for recommending that we update the plasmid reference data.

We made the following revisions:

(1) We replaced the 2021 release with the PLSDB 2025 update released online in November 2024, containing 72,360 plasmid records.

(2) We repeated identifier cleaning and redundancy removal, regenerated the data partitions, retrained CamFi, and updated all plasmid results and their interpretation.

**Revised text in the Datasets section:**

For plasmid sequences, we used the PLSDB 2025 update released online in November 2024 [50]. This release contains 72,360 plasmid records collected from RefSeq, GenBank, and the literature. After downloading the updated release, we repeated identifier cleaning and sequence deduplication, regenerated the training, validation, and test partitions, and retrained CamFi with the updated plasmid dataset.

**Revised text in the Plasmid Results:**

Across the five class-proportion settings, geNomad achieved a mean recall of 90.56%, CamFi achieved 90.30%, and PlasFlow achieved 75.96%. The overall recall difference was supported by one-way ANOVA. Pairwise Welch's t-tests yielded p = 0.885 for CamFi versus geNomad and p = 1.04e-03 for CamFi versus PlasFlow. For F1 score, CamFi achieved the highest mean of 94.13%, compared with 86.87% for geNomad and 69.90% for PlasFlow. The overall F1 difference yielded p = 0.0186 by one-way ANOVA; pairwise tests yielded p = 0.211 for CamFi versus geNomad and p = 0.0319 for CamFi versus PlasFlow.

Classification became more difficult as the proportion of plasmids decreased. When non-plasmid sequences accounted for 90% of the benchmark, CamFi retained an F1 score of 89.47%, whereas PlasFlow declined to 45.71%, a difference of 43.76 percentage points. These results indicate that CamFi was less sensitive to class-composition changes in the present benchmark; however, the statistical results above are based on five composition settings and should not be interpreted as evidence of universal superiority across independent datasets.

Fig. 6. Updated plasmid classification results

**Response to Reviewer #2**

***Comment 1:*** *DeepMicroClass should be discussed because the codon/base-path fusion is not itself conceptually new.*

Response: Thank you for identifying DeepMicroClass as an important related unified classifier.

We made the following revisions:

(1) We added DeepMicroClass to the Introduction and explicitly avoided claiming conceptual novelty for multi-input sequence classification.

(2) We distinguish CamFi by its parallel multi-scale nucleotide pathways and attention-based feature fusion and report a direct four-class benchmark comparison.

**Revised text in the Introduction:**

In metagenomics, although prokaryotic sequences are relatively tractable for classification, the precise delineation of viral, plasmid, and eukaryotic sequences remains challenging. Unified multi-class classification has previously been explored by DeepMicroClass [53], which predicts prokaryotes, eukaryotes, plasmids, and host-associated viral groups using deep neural models, and by 4CAC [54], which performs four-way classification of prokaryotes, viruses, plasmids, and microeukaryotes using length-adjusted XGBoost models followed by assembly-graph refinement. CamFi is therefore not presented as the first unified four-class classifier. Its contribution is a sequence-based architecture that integrates overlapping nucleotide-triplet patterns with multi-scale feature extraction and attention-based fusion to maintain stable classification across contig lengths.

**Revised text in the Four-class Benchmark Results:**

To compare CamFi with existing unified classifiers under an identical four-class setting, we evaluated CamFi, DeepMicroClass, and the sequence-based XGBoost stage of 4CAC (4CAC-XGBoost) on the balanced benchmark described above. Macro-averaged F1 was used as the common evaluation metric, as shown in Fig. 8.

CamFi achieved the highest macro-F1 score of 93.77%, followed by DeepMicroClass at 90.03% and 4CAC-XGBoost at 64.66%. The overall comparison across the five matched contig-length strata yielded p = 6.74e-03. Thus, CamFi exceeded DeepMicroClass by 3.74 percentage points and 4CAC-XGBoost by 29.11 percentage points on this benchmark.

**Revised text in the Discussion:**

Across the evaluated benchmarks, CamFi combined strong general four-class performance with task-dependent differences relative to specialized methods. On the balanced four-class benchmark, CamFi achieved a macro-F1 of 93.77%, compared with 90.03% for DeepMicroClass and 64.66% for the sequence-based XGBoost stage of 4CAC. In contrast, on the CAMI II virus benchmark, CamFi ranked second by aggregate F1 and remained below PPR-Meta. These results position CamFi as an extension of existing unified classification approaches rather than the first method of its kind. They support its utility under the present benchmark conditions without implying universal superiority across datasets or task-specific classifiers.

Fig. 8. Direct comparison with DeepMicroClass and 4CAC-XGBoost

***Comment 2:*** *The statistical treatment is inconsistent because the plasmid and CAMI II comparisons report point estimates without variance or significance testing.*

Response: Thank you for identifying the inconsistency in our original statistical reporting.

We made the following revisions:

(1) For the plasmid experiment, we added one-way ANOVA and pairwise Welch tests across the five matched class-composition settings and tempered the interpretation because these settings are not independent biological datasets.

(2) For CAMI II, only one aggregate result is available for each external method; because there is no replicated cross-method comparison unit, we explicitly report the ranking as descriptive and do not claim inferential significance.

**Revised text in the Plasmid Results:**

Across the five class-proportion settings, geNomad achieved a mean recall of 90.56%, CamFi achieved 90.30%, and PlasFlow achieved 75.96%. The overall recall difference was supported by one-way ANOVA. Pairwise Welch's t-tests yielded p = 0.885 for CamFi versus geNomad and p = 1.04e-03 for CamFi versus PlasFlow. For F1 score, CamFi achieved the highest mean of 94.13%, compared with 86.87% for geNomad and 69.90% for PlasFlow. The overall F1 difference yielded p = 0.0186 by one-way ANOVA; pairwise tests yielded p = 0.211 for CamFi versus geNomad and p = 0.0319 for CamFi versus PlasFlow.

Classification became more difficult as the proportion of plasmids decreased. When non-plasmid sequences accounted for 90% of the benchmark, CamFi retained an F1 score of 89.47%, whereas PlasFlow declined to 45.71%, a difference of 43.76 percentage points. These results indicate that CamFi was less sensitive to class-composition changes in the present benchmark; however, the statistical results above are based on five composition settings and should not be interpreted as evidence of universal superiority across independent datasets.

**Revised text in the CAMI II Results:**

On the CAMI II marine dataset, CamFi achieved a weighted F1 of 82.65%, ranking second among six methods based on reported aggregate metrics. Because only one aggregate result was available for each method, no valid cross-method significance test could be performed; the ranking is therefore descriptive.

Fig. 6. Plasmid comparison with overall and pairwise statistical results

Fig. 7. Descriptive aggregate comparison on CAMI II

***Comment 3:*** *The conclusions should reflect that CamFi trails PPR-Meta on the virus benchmark and should avoid overly broad generalization claims.*

Response: Thank you for emphasizing the need to report the virus benchmark ranking accurately and to limit the scope of our conclusions.

We made the following revisions:

(1) We now state explicitly that CamFi ranked second behind PPR-Meta on CAMI II.

(2) We limit all broader claims to the evaluated datasets and benchmark conditions and distinguish numerical ranking from universal superiority.

**Revised text in the CAMI II Results:**

On the CAMI II marine dataset, CamFi achieved a weighted F1 of 82.65%, ranking second among six methods based on reported aggregate metrics. Because only one aggregate result was available for each method, no valid cross-method significance test could be performed; the ranking is therefore descriptive.

**Revised text in the Discussion:**

Across the evaluated benchmarks, CamFi combined strong general four-class performance with task-dependent differences relative to specialized methods. On the balanced four-class benchmark, CamFi achieved a macro-F1 of 93.77%, compared with 90.03% for DeepMicroClass and 64.66% for the sequence-based XGBoost stage of 4CAC. In contrast, on the CAMI II virus benchmark, CamFi ranked second by aggregate F1 and remained below PPR-Meta. These results position CamFi as an extension of existing unified classification approaches rather than the first method of its kind. They support its utility under the present benchmark conditions without implying universal superiority across datasets or task-specific classifiers.

**Revised text in the Conclusion:**

Systematic evaluation demonstrates that CamFi achieves strong four-class classification performance under the tested community compositions. It attained the highest mean F1 in the eukaryotic chromosome and plasmid comparisons, although its difference from TIARA in the eukaryotic comparison was not statistically significant. On CAMI II, CamFi ranked second by aggregate F1 behind PPR-Meta and ahead of the other four evaluated virus classifiers. In the balanced four-class benchmark, CamFi achieved 93.77% macro-F1, compared with 90.03% for DeepMicroClass and 64.66% for 4CAC-XGBoost. Together with its stable performance across contig-length thresholds, these results support CamFi as a general-purpose classifier under the present benchmark conditions.

Fig. 7. Aggregate CAMI II comparison showing CamFi behind PPR-Meta

**Response to Additional Journal Requirements**

***Journal Requirement 1:*** *Please ensure that the manuscript meets PLOS ONE style requirements, including those for file naming.*

Response: Thank you for providing the PLOS ONE formatting and file-naming requirements.

We made the following revisions:

(1) We prepared separate marked and unmarked manuscripts and named all submission files consistently with the manuscript ID.

(2) Figures are named Fig1.tif-Fig8.tif and Supporting Information tables are named S1_Table.csv-S8_Table.csv.

**Revised text in the submission package:**

PONE-D-26-25179_Revised_Manuscript_with_Track_Changes.docx

PONE-D-26-25179_Manuscript.docx

Fig1.tif-Fig8.tif; S1_Table.csv-S8_Table.csv.

***Journal Requirement 2:*** *Please state what role the funders took in the study and include the amended Role of Funder statement in the cover letter.*

Response: Thank you for requesting clarification of the funders' roles.

We made the following revisions:

(1) We added the standard no-role statement for the grant funders to the Cover Letter.

(2) We clarified that Kelamayi Carbon Neutral Network Technology Co., Ltd. provided in-kind computational resources only and had no role in the scientific or publication decisions.

**Revised text in the Cover Letter:**

The funders had no role in study design, data collection and analysis, the decision to publish, or preparation of the manuscript. Kelamayi Carbon Neutral Network Technology Co., Ltd. provided in-kind computational resources only and had no role in study design, data collection and analysis, the decision to publish, or preparation of the manuscript.

***Journal Requirement 3:*** *Please remove funding-related text from the manuscript and provide the amended Funding Statement in the cover letter.*

Response: Thank you for clarifying that funding information must not appear in the manuscript Acknowledgements.

We made the following revisions:

(1) We removed the funding and company-support text and the Acknowledgements heading from both manuscript versions.

(2) We placed the complete amended Funding Statement in the Cover Letter for correction of the online submission record.

**Revised text in the Cover Letter:**

This work was supported by the Shanghai Natural Science Foundation (Grant No. 24ZR1421600), the National Natural Science Foundation of China (Grant No. 61802246), and in-kind computational resources provided by Kelamayi Carbon Neutral Network Technology Co., Ltd.

***Journal Requirement 4:*** *The Data Availability Statement should provide the repository name and a direct link to the underlying data.*

Response: Thank you for requesting a direct and complete repository statement.

We made the following revisions:

(1) We identified the CamFi GitHub repository and added its direct URL.

(2) We specified that it contains code, final model weights, benchmark datasets, SI Tables S1-S8, Mash workflows, the environment file, and third-party dataset download scripts.

**Revised text in the Data Availability section:**

The code, final model weights, benchmark datasets, Supplementary Information (SI) Tables S1-S8, Mash redundancy-removal workflows, environment file, and scripts for downloading third-party datasets are openly available in the CamFi GitHub repository: https://github.com/Milanio-dev/CamFi. Third-party source datasets remain subject to the terms of their original providers.

***Journal Requirement 5:*** *Please remove figures from the manuscript and upload the individual TIFF/EPS image files separately.*

Response: Thank you for specifying the required figure-submission format.

We made the following revisions:

(1) We removed all embedded figures from the two submission manuscripts while retaining their captions.

(2) We exported Figures 1-8 as individually named, 300-dpi TIFF files for separate upload.

**Revised text in the submission package:**

The separate figure files are reproduced below and supplied as Fig1.tif-Fig8.tif.

|   Fig. 1. Structure of 1D-DRCN |   Fig. 2. Structure of CamFi |
| --- | --- |
|   Fig. 3. Sensitivity analysis of hyperparameters on the validation set F1 score |   Fig. 4. Effect of sequence-length filtering thresholds on classification performance |
|   Fig. 5. Comparison of methods for classifying eukaryotic sequence fragments |   Fig. 6. Comparison of recall and F1 score for plasmid sequence classification |
|   Fig. 7. Aggregate accuracy and F1 score on the CAMI II dataset |   Fig. 8. Macro-F1 comparison on the balanced four-class benchmark |

**Figures 1-8 are supplied as separate 300-dpi TIFF files.**

***Journal Requirement 6:*** *Please include captions for Supporting Information files at the end of the manuscript and update the in-text citations accordingly.*

Response: Thank you for drawing our attention to the Supporting Information citation and caption requirements.

We made the following revisions:

(1) We added in-text citations to S1-S8 Tables at the relevant Methods and Results passages.

(2) We added a Supporting Information section containing captions for all eight files at the end of both manuscript versions.

**Revised text in the Supporting Information section:**

S1 Table. Complete hyperparameter grid-search results and validation metrics.

S2 Table. Precision, recall, and macro-F1 results across contig-length thresholds and random seeds.

S3 Table. Eukaryotic chromosome classification metrics across five class-composition settings.

S4 Table. Plasmid classification metrics across five class-composition settings.

S5 Table. Aggregate method comparison on the CAMI II marine dataset.

S6 Table. Sample-level CamFi metrics on the CAMI II marine dataset.

S7 Table. Macro-F1 comparison of CamFi, DeepMicroClass, and 4CAC-XGBoost on the balanced four-class benchmark.

S8 Table. Length-stratified macro-F1 results on the balanced four-class benchmark.

***Journal Requirement 7:*** *Please ensure that the grant information in the Funding Information and Financial Disclosure sections matches.*

Response: Thank you for identifying the inconsistency between the two online funding fields.

We made the following revisions:

(1) We provide one amended Funding Statement with the same two grant numbers and the same company name for use in both online fields.

(2) The statement is reproduced in the Cover Letter so that the journal can update the submission record consistently.

**Revised text in the Cover Letter:**

This work was supported by the Shanghai Natural Science Foundation (Grant No. 24ZR1421600), the National Natural Science Foundation of China (Grant No. 61802246), and in-kind computational resources provided by Kelamayi Carbon Neutral Network Technology Co., Ltd.

***Journal Requirement 8:*** *Please evaluate any reviewer-recommended publications and cite them only if they are relevant.*

Response: Thank you for clarifying that reviewer-recommended references should be evaluated for relevance rather than cited automatically.

We made the following revisions:

(1) We evaluated the suggested related methods and cited DeepMicroClass and 4CAC because both directly address unified metagenomic contig classification.

(2) We also replaced the outdated PLSDB citation with the relevant PLSDB 2025 update.

**Revised text in the Introduction:**

In metagenomics, although prokaryotic sequences are relatively tractable for classification, the precise delineation of viral, plasmid, and eukaryotic sequences remains challenging. Unified multi-class classification has previously been explored by DeepMicroClass [53], which predicts prokaryotes, eukaryotes, plasmids, and host-associated viral groups using deep neural models, and by 4CAC [54], which performs four-way classification of prokaryotes, viruses, plasmids, and microeukaryotes using length-adjusted XGBoost models followed by assembly-graph refinement. CamFi is therefore not presented as the first unified four-class classifier. Its contribution is a sequence-based architecture that integrates overlapping nucleotide-triplet patterns with multi-scale feature extraction and attention-based fusion to maintain stable classification across contig lengths.

**Revised text in the References:**

[50] Molano, L.-A.G., Hirsch, P., Hannig, M., Müller, R. and Keller, A. (2025) The PLSDB 2025 update: enhanced annotations and improved functionality for comprehensive plasmid research. Nucleic Acids Research, 53, D189-D196. https://doi.org/10.1093/nar/gkae1095.

[53] Hou, S., Tang, T., Cheng, S., Liu, Y., Xia, T., Chen, T., Fuhrman, J.A. and Sun, F. (2024) DeepMicroClass sorts metagenomic contigs into prokaryotes, eukaryotes and viruses. NAR Genomics and Bioinformatics, 6, lqae044.

[54] Pu, L. and Shamir, R. (2024) 4CAC: 4-class classifier of metagenome contigs using machine learning and assembly graphs. Nucleic Acids Research, 52, e94.
