# Supplementary material for: Codon-aware multi-scale feature fusion for metagenomic sequence classification
Source: PLoS One. 2026 Oct 5;21(10):e0359090. doi: 10.1371/journal.pone.0359090 (PMC13637925; doi:10.1371/journal.pone.0359090)
Supplement: Peer review history

# Peer review: Decision Letter 0

**DOI:** 10.1371/journal.pone.0359090.r001

**Authors:** Alfonso Esposito

8 Jul 2026

Dear Dr. Deng,

Thank you for submitting your manuscript to PLOS ONE. After careful consideration, we feel that it has merit but does not fully meet PLOS ONE’s publication criteria as it currently stands. Therefore, we invite you to submit a revised version of the manuscript that addresses the points raised during the review process.

==============================

**ACADEMIC EDITOR:**

The paper is potentially interesting, however it requires a deep revision. In light of the contrasting opinion of the two reviewers, I recommend major revisions. Please pay attention to the pitfalls highlighted by reviewer 1, which I consider pertinent and accurate. And revise the analyses and manuscript accordingly.

==============================

Please submit your revised manuscript by Sep 06 2026 11:59PM. If you will need more time than this to complete your revisions, please reply to this message or contact the journal office at plosone@plos.org. When you're ready to submit your revision, log on to https://www.editorialmanager.com/pone/ and select the 'Submissions Needing Revision' folder to locate your manuscript file.

Please include the following items when submitting your revised manuscript:

- A letter that responds to each point raised by the academic editor and reviewer(s). You should upload this letter as a separate file labeled 'Response to Reviewers'.
- A marked-up copy of your manuscript that highlights changes made to the original version. You should upload this as a separate file labeled 'Revised Manuscript with Track Changes'.
- An unmarked version of your revised paper without tracked changes. You should upload this as a separate file labeled 'Manuscript'.

If you would like to make changes to your financial disclosure, please include your updated statement in your cover letter. Guidelines for resubmitting your figure files are available below the reviewer comments at the end of this letter.

If applicable, we recommend that you deposit your laboratory protocols in protocols.io to enhance the reproducibility of your results. Protocols.io assigns your protocol its own identifier (DOI) so that it can be cited independently in the future. For instructions see: https://journals.plos.org/plosone/s/submission-guidelines#loc-laboratory-protocols. Additionally, PLOS ONE offers an option for publishing peer-reviewed Lab Protocol articles, which describe protocols hosted on protocols.io. Read more information on sharing protocols at https://plos.org/protocols?utm_medium=editorial-email&utm_source=authorletters&utm_campaign=protocols.

As the corresponding author, your ORCID iD is verified in the submission system and will appear in the published article. PLOS supports the use of ORCID, and we encourage all coauthors to register for an ORCID iD and use it as well. Please encourage your coauthors to verify their ORCID iD within the submission system before final acceptance, as unverified ORCID iDs will not appear in the published article. *Only* the individual author can complete the verification step; PLOS staff *cannot* verify ORCID iDs on behalf of authors.

We look forward to receiving your revised manuscript.

Kind regards,

Alfonso Esposito, PhD

Academic Editor

PLOS One

Journal Requirements:

When submitting your revision, we need you to address these additional requirements.

1.Please ensure that your manuscript meets PLOS ONE's style requirements, including those for file naming. The PLOS ONE style templates can be found at

https://journals.plos.org/plosone/s/file?id=wjVg/PLOSOne_formatting_sample_main_body.pdf and

https://journals.plos.org/plosone/s/file?id=ba62/PLOSOne_formatting_sample_title_authors_affiliations.pdf

2. Thank you for stating the following financial disclosure:

“Shanghai Natural Science Foundation (Grant No. 24ZR1421600)

the National Natural Science Foundation of China (Grant No. 61802246)

Kelamayi Carbon Neutral network technology co., Ltd.”

Please state what role the funders took in the study.  If the funders had no role, please state: "The funders had no role in study design, data collection and analysis, decision to publish, or preparation of the manuscript."

If this statement is not correct you must amend it as needed.

Please include this amended Role of Funder statement in your cover letter; we will change the online submission form on your behalf.

3. Thank you for stating the following in the Acknowledgments Section of your manuscript:

“This study was supported by the Shanghai Natural Science Foundation (Grant No. 24ZR1421600) and the National Natural Science Foundation of China (Grant No. 61802246).”

We note that you have provided funding information that is not currently declared in your Funding Statement. However, funding information should not appear in the Acknowledgments section or other areas of your manuscript. We will only publish funding information present in the Funding Statement section of the online submission form.

Please remove any funding-related text from the manuscript and let us know how you would like to update your Funding Statement. Currently, your Funding Statement reads as follows:

“Shanghai Natural Science Foundation (Grant No. 24ZR1421600)

the National Natural Science Foundation of China (Grant No. 61802246)

Kelamayi Carbon Neutral network technology co., Ltd.”

Please include your amended statements within your cover letter; we will change the online submission form on your behalf.

4. Please note that your Data Availability Statement is currently missing the repository name and/or the DOI/accession number of each dataset OR a direct link to access each database. If your manuscript is accepted for publication, you will be asked to provide these details on a very short timeline. We therefore suggest that you provide this information now, though we will not hold up the peer review process if you are unable.

5. Please remove your figures from within your manuscript file, leaving only the individual TIFF/EPS image files, uploaded separately. These will be automatically included in the reviewers’ PDF.

6. Please include captions for your Supporting Information files at the end of your manuscript, and update any in-text citations to match accordingly. Please see our Supporting Information guidelines for more information: http://journals.plos.org/plosone/s/supporting-information.

7. We note that the grant information you provided in the ‘Funding Information’ and ‘Financial Disclosure’ sections do not match.

When you resubmit, please ensure that you provide the correct grant numbers for the awards you received for your study in the ‘Funding Information’ section.

8. If the reviewer comments include a recommendation to cite specific previously published works, please review and evaluate these publications to determine whether they are relevant and should be cited. There is no requirement to cite these works unless the editor has indicated otherwise.

[Note: HTML markup is below. Please do not edit.]

Reviewer's Responses to Questions

**Comments to the Author**

1. Is the manuscript technically sound, and do the data support the conclusions?

Reviewer #1: No

Reviewer #2: Yes

**********

2. Has the statistical analysis been performed appropriately and rigorously?

Reviewer #1: No

Reviewer #2: Yes

**********

3. Have the authors made all data underlying the findings in their manuscript fully available?

The PLOS Data policy

Reviewer #1: Yes

Reviewer #2: Yes

**********

4. Is the manuscript presented in an intelligible fashion and written in standard English?

Reviewer #1: No

Reviewer #2: Yes

**********

Reviewer #1: The paper describes a deep learning method to classify metagenomic contigs into prokaryotic based, eukaryotic based, plasmids, and viruses. The authors propose a One-Dimensional Dilated Residual Convolution Network (1D-DRCN) and a multi-head attention mechanism pathway to achieve this. According to the authors, the innovation lies in capturing genomic features across varying spatial scales; defined as long, medium, and short scales. The authors further mention that the short scale is capable of integrating codon usage bias to achieve high-accuracy classifications.

While the ambition and proposed mechanism is interesting, I find several issues with the manuscript. Please let me outline them:

1) Issues with terminology and protocol: My largest gripe in terms of terminology is with the usage of “Codon usage bias”. The authors mention codon usage bias in both the abstract, and the methods section. But the protocol they have adopted is definitely not analysing codon usage or codon usage biases.

The short pathway part of the CamFi architecture makes use of a “sliding window of 3bp”. The figure 2 mentions that they used a stride of 1. This would mean that the authors are looking at a continuous overlapping 3-mer and not non overlapping codons based on ORFs. This sliding window of 3-mer does not make CamFI codon aware.

Codon usage bias: This is a completely different idea altogether.

Most amino acids are encoded by multiple codons. If a sequence uses codons randomly, then all six codons for an amino acid (eg. Leucine) would be used equally. However, some bacteria prefer specific synonymous codons over others. Thus, "bias" occurs when an organism or sequence heavily favors one or two of those synonymous codons over the others.

I would recommend the authors look at how codon awareness was incorporated in PPR-Meta – which shows better prediction for viruses in their own tests. In future manuscripts I would recommend the authors use codon aware rather than codon usage bias.

2) “Long-scale” for promoter regions: I am not certain why the authors mention that 15bp is sufficient to recognize promoter regions. The 15 bp region might be sufficient to recognize ribosome binding sites or transcription factor binding motif, but extending it to also include promoter regions sounds hard to comprehend when most eukaryotic promoter regions are 100s to 1000s of bps in size with spacers in the middle. Even bacterial promoters are greater than 30bps.

Some kind of evidence for this recognition would be useful to back the claim.

3) Issues with training (Transcriptome vs metagenome) and why whokaryote might be underperforming: The authors make use of MMETSP transcriptomic data. However, this choice is not very well justified. The authors mention:

“For eukaryotic transcripts from MMETSP, given that some samples have incomplete taxonomic annotations and transcriptome assemblies are prone to misassemble errors, partitioning was performed according to MMETSP sample identifiers.”

and

“All data groups were partitioned at a ratio of 70% training, 15% validation, and 15% test sets.”

Transcriptomes consist entirely of dense, continuous protein-coding regions (exons). Metagenomics deals with raw environmental DNA characterized by expansive intergenic spacers and massive intronic regions.

This means that Whokaryote was also run on the transcript based data. But whokaryote makes use of intergenic distances to predict eukaryotic contigs. Applying this to a transcript based data is not valid. The authors should test CamFi on only whole metagenome and not include MMETSP data.

Using the MMETSP data for training and then testing it on the same dataset is also not valid. Even holding out sample IDs does not close the domain gap because the test set is still transcriptomic. This could be resolved by restricting the eukaryotic dataset strictly to whole-genome shotgun assemblies. The CAMI II benchmarking was limited to only prokaryotes and viruses. The authors do not mention any real/ simulated community benchmarking for eukaryotic metagenome samples.

4) Comparison against similar tools:

The claim “no existing method is capable of simultaneously discriminating among these four sequence categories within a unified framework” by the authors is just wrong.

The authors should test CamFI against similar tools like DeepMicroClass and 4CAC. A confusion matrix with their results would be an improved comparison along with F1 score.

5) Old plasmid reference dataset: The PLSDB version used is from 2021. There is a new version that is available that doubles the sequences from ~34k sequences to ~70K sequences in the 2024 version. It would be nice if the authors used this contemporary version to train/ test CamFi.

Reviewer #2: CamFi (Codon-aware Multi-scale Feature Fusion) REVIEW

Summary of the Key Results

The paper presents CamFi, a CNN classifying metagenomic contigs into prokaryotic chromosomes, eukaryotic chromosomes, plasmids, and viruses, using three parallel dilated pathways (long/medium-scale nucleotide, short-scale codon) fused via multi-head attention. On synthetic data, CamFi significantly outperforms Whokaryote and Tiara on eukaryotic chromosomes (ANOVA p<0.01) and beats geNomad/PlasFlow on plasmid F1 (93.40%), staying far more robust than PlasFlow under heavy noise. On the CAMI II marine dataset, CamFi ranks second of six virus classifiers (F1 80.75%), behind PPR-Meta but ahead of VirSorter2, geNomad, DeepVirFinder and VIBRANT. Length-filtering analysis shows macro-F1 rising from 0.905 (2kb) to 0.944 (10kb), with growing variance at longer thresholds.

Originality and Significance

The codon-path/base-path fusion is not conceptually new (PPR-Meta, DeepMicroClass, this last one not mentioned and should probably be at least reported in the conclusions); CamFi's contribution is splitting the nucleotide branch into two dilated sub-scales and using attention rather than concatenation for fusion. This is a reasonable, incremental refinement, with solid biological intuition that the 2 sliding windows could capture different biological elements. While CamFi's clearest wins are against weaker baselines (Whokaryote, PlasFlow), the performance on other task are comparable and the result of an unbiased methodological comparison.

Data and Methodology

My understanding of sinthetic data is limited and I can only comment on being the standard in all the papers I have read on the topic, with good reasoning behind it in the text

Appropriate Use of Statistics

Statistical approaches are solid but inconsistent across subsections. The eukaryotic chromosome comparison is done well, with ANOVA plus pairwise t-tests. The plasmid and CAMI II virus comparisons report only point estimates, with no variance or significance testing. While the results on the eukaryotic part are the main results and it’s reasonable for the authors to be more complete in that side, at least some basic variance reporting (even just in the text) would be appreaciated.

Conclusion: Robustness, Validity and Reliability

The eukaryotic chromosome result is well supported statistically and is the strongest evidence of validity in the paper. The plasmid and noise-robustness results are directionally convincing but rest on point estimates rather than tested differences, so they should be read as promising rather than confirmed. The virus result is honestly reported and reliable, but shows CamFi trailing the architecturally closest baseline (PPR-Meta), which limits how strongly the multi-scale/attention design can be said to generalize. Overall the work is robust for two of its four classes and plausible for the others, which is a fair basis for publication.

Clarity and Context

The manuscript is clear and well organized. It follow the convention I have seen used in the field and it contextualize properly when need when technical elements are essential.

**********

what does this mean?). If published, this will include your full peer review and any attached files.

If you choose “no”, your identity will remain anonymous but your review may still be made public.

**Do you want your identity to be public for this peer review?** For information about this choice, including consent withdrawal, please see our Privacy Policy

Reviewer #1: No

Reviewer #2: No

**********

[NOTE: If reviewer comments were submitted as an attachment file, they will be attached to this email and accessible via the submission site. Please log into your account, locate the manuscript record, and check for the action link "View Attachments". If this link does not appear, there are no attachment files.]

To ensure your figures meet our technical requirements, please review our figure guidelines: https://journals.plos.org/plosone/s/figures

You may also use PLOS’s free figure tool, NAAS, to help you prepare publication quality figures: https://journals.plos.org/plosone/s/figures#loc-tools-for-figure-preparation.

NAAS will assess whether your figures meet our technical requirements by comparing each figure against our figure specifications.

# Author comment: Author response to Decision Letter 1

**DOI:** 10.1371/journal.pone.0359090.r002

3 Sep 2026

Dear Editor and Reviewers,

Thank you for your careful and constructive comments. We have revised the manuscript accordingly and provided detailed point-by-point responses. All changes are highlighted in the marked manuscript and incorporated into the clean version. The revised figures, Supporting Information, and publicly available data and code have also been updated.

Sincerely,

The Authors

Attachment Submitted filename: PONE-D-26-25179_Response_to_Reviewers.docx

# Editor report: Decision Letter 1

**DOI:** 10.1371/journal.pone.0359090.r003

**Authors:** Alfonso Esposito

10 Sep 2026

Codon-Aware Multi-Scale Feature Fusion for Metagenomic Sequence Classification

PONE-D-26-25179R1

Dear Dr. Deng,

We’re pleased to inform you that your manuscript has been judged scientifically suitable for publication and will be formally accepted for publication once it meets all outstanding technical requirements.

Within one week, you’ll receive an e-mail detailing the required amendments. When these have been addressed, you’ll receive a formal acceptance letter and your manuscript will be scheduled for publication.

An invoice will be generated when your article is formally accepted. Please note, if your institution has a publishing partnership with PLOS and your article meets the relevant criteria, all or part of your publication costs will be covered. Please make sure your user information is up-to-date by logging into Editorial Manager at Editorial Manager® and clicking the ‘Update My Information' link at the top of the page. For questions related to billing, please contact billing support.

If your institution or institutions have a press office, please notify them about your upcoming paper to help maximize its impact. If they’ll be preparing press materials, please inform our press team as soon as possible -- no later than 48 hours after receiving the formal acceptance. Your manuscript will remain under strict press embargo until 2 pm Eastern Time on the date of publication. For more information, please contact onepress@plos.org.

Kind regards,

Alfonso Esposito, PhD

Academic Editor

PLOS One

Additional Editor Comments (optional):

Reviewers' comments:

# Editor report: Acceptance letter

**DOI:** 10.1371/journal.pone.0359090.r004

**Authors:** Alfonso Esposito

PONE-D-26-25179R1

PLOS One

Dear Dr. Deng,

I'm pleased to inform you that your manuscript has been deemed suitable for publication in PLOS One. Congratulations! Your manuscript is now being handed over to our production team.

At this stage, our production department will prepare your paper for publication. This includes ensuring the following:

* All references, tables, and figures are properly cited

* All relevant supporting information is included in the manuscript submission,

* There are no issues that prevent the paper from being properly typeset

You will receive further instructions from the production team, including instructions on how to review your proof when it is ready. Please keep in mind that we are working through a large volume of accepted articles, so please give us a few days to review your paper and let you know the next and final steps.

Lastly, if your institution or institutions have a press office, please let them know about your upcoming paper now to help maximize its impact. If they'll be preparing press materials, please inform our press team within the next 48 hours. Your manuscript will remain under strict press embargo until 2 pm Eastern Time on the date of publication. For more information, please contact onepress@plos.org.

You will receive an invoice from PLOS for your publication fee after your manuscript has reached the completed accept phase. If you receive an email requesting payment before acceptance or for any other service, this may be a phishing scheme. Learn how to identify phishing emails and protect your accounts at https://explore.plos.org/phishing.

If we can help with anything else, please email us at customercare@plos.org.

Thank you for submitting your work to PLOS One and supporting open access.

Kind regards,

PLOS One Editorial Office Staff

on behalf of

Prof. Alfonso Esposito

Academic Editor

PLOS One
